# The complexity of divisibility

**DOI:** 10.1016/j.laa.2016.03.041

**Published:** 2016-09-01

**Authors:** Johannes Bausch, Toby Cubitt

**Affiliations:** a DAMTP, Centre for Mathematical Sciences, University of Cambridge, Wilberforce Road, Cambridge CB3 0WB, UK; b Department of Computer Science, University College London, Gower Street, London WC1E 6BT, UK

**Keywords:** 60-08, 81-08, 68Q30, Stochastic matrices, cptp maps, Probability distributions, Divisibility, Decomposability, Complexity theory

## Abstract

We address two sets of long-standing open questions in linear algebra and probability theory, from a computational complexity perspective: stochastic matrix divisibility, and divisibility and decomposability of probability distributions. We prove that finite divisibility of stochastic matrices is an NP-complete problem, and extend this result to nonnegative matrices, and completely-positive trace-preserving maps, i.e. the quantum analogue of stochastic matrices. We further prove a complexity hierarchy for the divisibility and decomposability of probability distributions, showing that finite distribution divisibility is in P, but decomposability is NP-hard. For the former, we give an explicit polynomial-time algorithm. All results on distributions extend to weak-membership formulations, proving that the complexity of these problems is robust to perturbations.

## 1 Introduction and overview

People have pondered divisibility questions throughout most of Western science and philosophy. Perhaps the earliest written mention of divisibility is in *Aristotle's Physics* in 350 BC, in the form of the *Arrow paradox*—one of *Zeno of Elea's* paradoxes (ca. 490–430 BC). Aristotle's lengthy discussion of divisibility (he devotes an entire chapter to the topic) was motivated by the same basic question as more modern divisibility problems in mathematics: can the behaviour of an object—physical or mathematical—be subdivided into smaller parts?

For example, given a description of the evolution of a system over some time interval *t*, what can we say about its evolution over the time interval t/2? If the system is stochastic, this question finds a precise formulation in the *divisibility problem* for stochastic matrices [19]: given a stochastic matrix **P**, can we find a stochastic matrix **Q** such that $P=Q^{2}$?

This question has many applications. For example, in information theory stochastic matrices model noisy communication channels, and divisibility becomes important in *relay coding*, when signals must be transmitted between two parties where direct end-to-end communication is not available [23]. Another direct use is in the analysis of chronic disease progression [3], where the transition matrix is based on sparse observations of patients, but finer-grained time-resolution is needed. In finance, changes in companies' credit ratings can be modelled using discrete time Markov chains, where rating agencies provide a transition matrix based on annual estimates—however, for valuation or risk analysis, a transition matrix for a much shorter time periods needs to be inferred [17].

We can also ask about the evolution of the system for *all* times up to time *t*, i.e. whether the system can be described by some continuous evolution. For stochastic matrices, this has a precise formulation in the *embedding problem*: given a stochastic matrix **P**, can we find a generator **Q** of a continuous-time Markov process such that P=exp⁡(Qt)? The embedding problem seems to date back further still, and was already discussed by Elfving in 1937 [10]. Again, this problem occurs frequently in the field of systems analysis, and in analysis of experimental time-series snapshots [7], [22], [27].

Many generalizations of these divisibility problems have been studied in the mathematics and physics literature. For example, the question of square-roots of (entry-wise) nonnegative matrices is an old open problem in matrix analysis [24]: given an entry-wise nonnegative matrix **M**, does it have an entry-wise nonnegative square-root? In quantum mechanics, the analogue of a stochastic matrix is a completely-positive trace preserving (cptp) map, and the corresponding divisibility problem asks: when can a cptp map **T** be decomposed as T=R∘R, where **R** is itself cptp? The continuous version of this, whether a cptp can be embedded into a completely-positive semi-group, is sometimes called the *Markovianity problem* in physics [8]—the latter again has applications to subdivision coding of quantum channels in quantum information theory [26].

Instead of dynamics, we can also ask whether the description of the static state of a system can be subdivided into smaller, simpler parts. Once again, probability theory provides a rich source of such problems. The most basic of these is the classic topic of divisible distributions: given a random variable *X*, can it be decomposed into X=Y+Z where Y,Z are some other random variables? What if *Y* and *Z* are identically distributed? If we instead ask for a decomposition into infinitely many random variables, this becomes the question of whether a distribution is infinitely divisible.

In this work, we address two of the most long-standing open problems on divisibility: divisibility of stochastic matrices, and divisibility and decomposability of probability distributions. We also extend our results to divisibility of nonnegative matrices and completely positive maps. Surprisingly little is known about the divisibility of stochastic matrices. Dating back to 1962 [19], the most complete characterization remains for the case of a 2×2 stochastic matrix [14]. The infinite divisibility problem has recently been solved [8], but the finite case remains an open problem. Divisibility of random variables, on the other hand, is a widely-studied topic. Yet, despite first results dating back as far as 1934 [5], no general method of answering whether a random variable can be written as the sum of two or more random variables—whether distributed identically, or differently—is known.

We focus on the computational complexity of these divisibility problems. In each case, we show which of the divisibility problems have efficient solutions—for these, we give an explicit efficient algorithm. For all other cases, we prove reductions to the famous P=NP-conjecture, showing that those problems are NP-hard. This essentially implies that—unless P=NP—the geometry of the corresponding divisible and non-divisible is highly complex, and these sets have no simple characterization beyond explicit enumeration. In particular, this shows that any future concrete classification of these NP-hard problems will be at least as hard as answering P=NP.

The following theorems summarize our main results on maps. Precise formulations and proofs can be found in section 2.

Theorem 1 *Given a stochastic matrix* **P***, deciding whether there exists a stochastic matrix* **Q** *such that* $P=Q^{2}$ *is* NP*-complete.*

Theorem 2 *Given a* cptp *map* **B***, deciding whether there exists a* cptp *map* **A** *such that* B=A∘A *is* NP*-complete.*

In fact, the last two theorems are strengthenings of the following result.

Theorem 3 *Given a nonnegative matrix* **M***, deciding whether there exists a nonnegative matrix* **N** *such that* $M=N^{2}$ *is* NP*-complete.*

The following theorems summarize our main results on distributions. Precise formulations and proofs can be found in section 3.

Theorem 4 *Let X be a finite discrete random variable. Deciding whether X is n-divisible—i.e. whether there exists a random variable Y such that* $X=\sum_{i=1}^{n}Y$*—is in* P*.*

Theorem 5 *Let X be a finite discrete random variable, and* ϵ>0*. Deciding whether there exists a random variable Y ϵ-close to X such that Y is n-divisible, or that there exists such a Y that is nondivisible, is contained in* P*.*

Theorem 6 *Let X be a finite discrete random variable. Deciding whether X is decomposable—i.e. whether there exist random variables* Y,Z *such that* X=Y+Z*—is* NP*-complete.*

Theorem 7 *Let X be a finite discrete random variable, and* ϵ>0*. Deciding whether there exists a random variable Y ϵ-close to X such that Y is decomposable, or that there exists such an ϵ-close Y that is indecomposable, is* NP*-complete.*

It is interesting to contrast the results on maps and distributions. In the case of maps, the homogeneous 2-divisibility problems are already NP-hard, whereas finding an inhomogeneous decomposition is straightforward. For distributions, on the other hand, the homogeneous divisibility problems are efficiently solvable to all orders, but becomes NP-hard if we relax it to the inhomogeneous decomposability problem.

This difference is even more pronounced for infinite divisibility. The infinite divisibility problem for maps is NP-hard (shown in [8]), whereas the infinite divisibility and decomposability problems for distributions are computationally trivial, since indivisible and indecomposable distributions are both dense—see section 3.5.8 and 3.4.5.

The paper is divided into two parts. We first address stochastic matrix and cptp divisibility in section 2, obtaining results on entry-wise positive matrix roots along the way. Divisibility and decomposability of probability distributions is addressed in section 3. In both sections, we first give an overview of the history of the problem, stating previous results and giving precise definitions of the problems. We introduce the necessary notation at the beginning of each section, so that each section is largely self-contained.

## 2 CPTP and stochastic matrix divisibility

### 2.1 Introduction

Mathematically, subdividing Markov chains is known as the *finite divisibility* problem. The simplest case is the question of finding a stochastic root of the transition matrix (or a cptp root of a cptp map in the quantum setting), which corresponds to asking for the evolution over half of the time interval. While the question of divisibility is rather simple to state mathematically, it is not clear a priori whether a stochastic matrix root for a given stochastic matrix exists at all. Historically, this has been a long-standing open question, dating back to at least 1962 [19]. Matrix roots were also suggested early on in other fields, such as economics and general trade theory, at least as far back as 1967 [31], to model businesses and the flow of goods. Despite this long history, very little is known about the existence of stochastic roots of stochastic matrices. The most complete result to date is a full characterization of 2×2 matrices, as given for example in [14]. The authors mention that *“…it is quite possible that we have to deal with the stochastic root problem on a case-by-case basis.”* This already suggests that there might not be a simple mathematical characterization of divisible stochastic matrices—meaning one that is simpler than enumerating the exponentially many roots and checking each one for stochasticity.

There are similarly few results if we relax the conditions on the matrix normalization slightly, and ask for (entry-wise) nonnegative roots of (entry-wise) nonnegative matrices—for a precise formulation, see Definition 10, Definition 11. An extensive overview can be found in [24]. Following this long history of classical results, quantum channel divisibility recently gained attention in the quantum information literature. The foundations were laid in [33], where the authors first introduced the notion of *channel divisibility*. A divisible quantum channel is a cptp map that can be written as a nontrivial concatenation of two or more quantum channels.

A related question is to ask for the evolution under infinitesimal time steps, which is equivalent to the existence of a logarithm of a stochastic matrix (or cptp map) that generates a stochastic (resp. cptp) semi-group. Classically, the question is known as *Elfving's* problem or the *embedding problem*, and it seems to date back even further than the finite case, to 1937 [10]. In the language of Markov chains, this corresponds to determining whether a given stochastic matrix can be embedded into an underlying continuous time Markov chain. Analogously, infinite quantum channel divisibility—also known as the *Markovianity* condition for a cptp map—asks whether the dynamics of the quantum system can be described by a *Lindblad* master equation [21], [12]. The infinite divisibility problems in both the classical and quantum case were recently shown to be NP-hard [8]. Formulated as weak membership problems, these results imply that it is NP-hard to extract dynamics from experimental data [7].

However, while related, it is not at all clear that there exists a reduction of the finite divisibility question to the case of infinite divisibility. In fact, mathematically, the infinite divisibility case is a special case of finite divisibility, as a stochastic matrix is infinitely divisible if and only if it admits an *n*^th^ root for all n∈N [19].

The finite divisibility problem for stochastic matrices is still an open question, as are the nonnegative matrix and cptp map divisibility problems. We will show that the question of existence of stochastic roots of a stochastic matrix is NP-hard. We also extend this result to (doubly) stochastic matrices, nonnegative matrices, and cptp maps.

We start out by introducing the machinery we will use to prove Theorem 1, Theorem 3 in section 2.2. A reduction from the quantum to the classical case can be found in section 2.4, from the nonnegative to the stochastic case in section 2.5 and the main result—in a mathematically rigorous formulation—is then presented as Theorem 20 in section 2.6.

### 2.2 Preliminaries

#### 2.2.1 Roots of matrices

In our study of matrix roots we restrict ourselves to the case of square roots. The more general case of *p*^th^ roots of matrices remains to be discussed. We will refer to square roots simply as roots. To be explicit, we state the following definition.

Definition 8 Let $M\in K^{d\times d}$, d∈N, K some field. Then we say that $R\in K^{d\times d}$ is a root of **M** if $R^{2}=M$. We denote the set of all roots of **M** with $\sqrt{M}$.

Following the theory of matrix functions—see for example [15]—we remark that in the case of nonsingular **M**, $\sqrt{M}$ is nonempty and can be expressed in Jordan normal form via $\sqrt{M}=ZJZ^{-1}$ for some invertible **Z**, where $J=\mathrm{diag}(J_{1}^{\pm },\ldots ,J_{m}^{\pm })$. Here $J_{i}^{\pm }$ denotes the ±-branch of the root function $f(x)=\sqrt{x}$ of the Jordan block corresponding to the *i*th eigenvalue $\lambda_{i}$,

$$J_{i}^{\pm }=\left(\begin{array}{cccc} \pm f(\lambda_{i}) & \pm f^{\prime }(\lambda_{i})/1! & \ldots & \pm f^{\left(m_{i}-1\right)}(\lambda_{i})/(m_{i}-1)!\\ 0 & \pm f(\lambda_{i}) & \ddots & \vdots \\ \vdots & \ddots & \ddots & \pm f^{\prime }(\lambda_{i})/1!\\ 0 & \ldots & 0 & \pm f(\lambda_{i}) \end{array}\right)\;\;.$$

If **M** is diagonalizable, **J** simply reduces to the canonical diagonal form $J=\mathrm{diag}(\pm \sqrt{\lambda_{1}},\ldots ,\pm \sqrt{\lambda_{m}})$.

If **M** is derogatory—i.e. there exist multiple Jordan blocks sharing the same eigenvalue *λ*—it has continuous families of so-called *nonprimary* roots $\sqrt{M}=ZUJU^{-1}Z^{-1}$, where **U** is an arbitrary nonsingular matrix that commutes with the Jordan normal form [U,J]=0.

We cite the following result from [16, Th. 2.6].

Theorem 9 **Classification of roots**

*Let* $M\in K^{d\times d}$ *have the Jordan canonical form* $Z\Lambda Z^{-1}$*, where* $\Lambda =\mathrm{diag}(J_{0},J_{1})$*, such that* $J_{0}$ *collects all Jordan blocks corresponding to the eigenvalue* 0*, and* $J_{1}$ *collects the remaining ones. Assume further that*

$$d_{i}:=\dim(\ker\;M^{i})-\dim(\ker\;M^{i-1})$$

*has the property that for all* $i\in N_{\geq 0}$*, no more than one element of the sequence satisfies* $d_{i}\in (2i,2(i+1))$*. Then* $\sqrt{M}=Z\sqrt{\Lambda }Z^{-1}$*, where* $\sqrt{\Lambda }=\mathrm{diag}(\sqrt{J_{0}},\sqrt{J_{1}})$*.*

For a given matrix, the classification gives the set of all roots. If **M** is a real matrix, a similar theorem holds and there exist various numerical algorithms for calculating real square roots, see for example [15].

#### 2.2.2 Roots of stochastic matrices

Remember the following two definitions.

Definition 10 A matrix $M\in K^{d\times d}$ is said to be nonnegative if $0\leq M_{ij}\;\forall i,j=0,\ldots ,d$.

Definition 11 A matrix $Q\in K^{d\times d}$ is said to be stochastic if it is nonnegative and $\sum_{k=1}^{d}Q_{ik}=1\;\forall i=0,\ldots ,d$.

In contrast to finding a general root of a matrix, very little is known about the existence of nonnegative roots of nonnegative matrices—or stochastic roots of stochastic matrices—if d≥3. For stochastic matrices and in the case d=2, a complete characterization can be given explicitly, and for d≥3, all real stochastic roots that are functions of the original matrix are known, as demonstrated in [14]. Further special classes of matrices for which a definite answer exists can be found in [16]. But even for d=3, the general case is still an open question—see [20, ch. 2.3] for details.

Indeed, a stochastic matrix may have no stochastic root, a primary or nonprimary root—or both. To make things worse, if a matrix has a *p*^th^ stochastic root, it might or might not have a *q*^th^ stochastic root if p∤q—*p* is not a divisor of *q*—, q>p or q∤p, q<p.

A related open problem is the inverse eigenspectrum problem, as described in the extensive overview in [9]. While the sets $\Omega_{n}\subset D$—denoting all the possible valid eigenvalues of an *n*-dimensional stochastic matrix—can be given explicitly, and hence also $\Omega_{n}^{p}$, almost nothing is known about the sets of valid eigenspectra. Any progress in this area might yield necessary conditions for the existence of stochastic roots.

In recent years, some approaches have been developed to approximate stochastic roots numerically, see the comments in [14, sec. 4]. Unfortunately, most algorithms are highly unstable and do not necessarily converge to a stochastic root. A direct method using nonlinear optimization techniques is difficult and depends heavily on the algorithm employed [20].

It remains an open question whether there exists an efficient algorithm that decides whether a stochastic matrix **Q** has a stochastic root.

*In this paper, we will prove that this question is* NP*-hard to answer.*

#### 2.2.3 The Choi isomorphism

For the results on cptp maps, we will need the following basic definition and results.

Definition 12 Let A:H⟶H be a linear map on $H=C^{d\times d}$. We say that **A** is positive if for all Hermitian and positive definite ρ∈H, Aρ is Hermitian and positive definite. It is said to be completely positive if $A⊗1_{n}$ is positive ∀n∈N.

A map **A** which is completely positive and trace-preserving—i.e. tr(Aρ)=trρ∀ρ∈H—is called a *completely positive trace-preserving* map, or short cptp map.

In contrast to positivity, complete positivity is easily characterized using the well-known *Choi–Jamiolkowski* isomorphism—cf. [4, Th. 2].

Remark 13 Let the notation be as in Definition 12 and pick a basis $e_{1},\ldots ,e_{d}$ of $C^{d}$. Then **A** is completely positive if and only if the *Choi* matrix

$$C_{A}:=(1_{d}⊗A)\Omega \Omega^{T}=\sum_{i,j=1}^{d}e_{i}e_{j}^{T}⊗A(e_{i}e_{j}^{T})$$

is positive semidefinite, where $\Omega :=\sum_{i=1}^{d}e_{i}⊗e_{i}$.

The condition of trace-preservation then translates to the following.

Remark 14 A map **A** is trace-preserving if and only if ${\mathrm{tr}}_{2}(C_{A})=1_{d}$, where ${\mathrm{tr}}_{2}$ denotes the partial trace over the second pair of indices.

### 2.3 Equivalence of computational questions

In the following we denote with *S* some arbitrary finite index set, not necessarily the same for all problems. We begin by defining the following decision problems.

Definition 15 **cptp Divisibility**

**Instance.**: cptp map $B\in Q^{d\times d}$.
**Question.**: Does there exist a cptp map $A:A^{2}=B$?

Definition 16 **cptp Root**

**Instance.**: Family of matrices ${\left(A_{s}\right)}_{s\in S}$ that comprises all the roots of a matrix **B**.
**Question.**: Does there exist an $s\in S:A_{s}$ is a cptp map?

Definition 17 **Stochastic Divisibility**

**Instance.**: Stochastic matrix $P\in Q^{d\times d}$.
**Question.**: Does there exist a stochastic matrix $Q:Q^{2}=P$?

Definition 18 **Stochastic Root**

**Instance.**: Family of matrices ${\left(Q_{s}\right)}_{s\in S}$ comprising all the roots of a matrix **P**.
**Question.**: Does there exist an $s\in S:Q_{s}$ stochastic?

Definition 19 **Nonnegative Root**

**Instance.**: Family of matrices ${\left(M_{s}\right)}_{s\in S}$ comprising all the roots of a matrix **N**, where all $M_{s}$ have at least one positive entry.
**Question.**: Does there exist an $s\in S:M_{s}$ nonnegative?

**Fig. 1:**
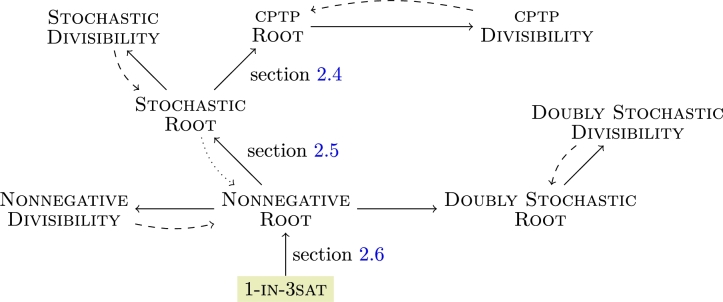
Complete chain of reduction for our programs. The dashed line between the Divisibility and Root problems hold for non-derogatory matrices, respectively. The dotted line between Stochastic Root and Nonnegative Root holds only for irreducible matrices. The doubly stochastic and nonnegative branch are included for completeness but not described in detail here—see Corollary 27.

Theorem 20 *The reductions as shown in* Fig. 1 *hold.*

Proof The implication Stochastic Divisibility⟵Stochastic Root needs one intermediate step. If **P** is not stochastic, the answer is negative. If it is stochastic, we can apply Stochastic Divisibility. The opposite direction holds for non-derogatory stochastic **P**: in this case we can enumerate all roots of **P** as a finite family which forms a valid instance for Stochastic Root.

The reduction Stochastic Root⟵Nonnegative Root can be resolved by Lemma 25 and Lemma 26—we construct a family of matrices ${\left(Q_{s}\right)}_{s\in S}$ that contains a stochastic root iff ${\left(M_{s}\right)}_{s\in S}$ contains a nonnegative root. The result then follows from applying Stochastic Root. If our stochastic matrix **P** is irreducible, then any nonnegative root $Q_{s^{\prime }}:Q_{s^{\prime }}^{2}=P$ is stochastic, and in that case Stochastic Root⟷Nonnegative Root—see [16, sec. 3] for details.

The link cptp Divisibility⟵cptp Root again needs the following intermediate step. If **A** is not cptp, the answer is negative. If it is cptp, then we can apply cptp Divisibility. Similarly, if **A** is non-derogatory, the reduction works in the opposite direction as well.

The direction cptp Root⟵Stochastic Root follows from Corollary 24. We start out with a family ${\left(Q_{s}\right)}_{s\in S}$ comprising all the roots of a stochastic matrix **P**. Then let ${\left(A_{s}:=\mathrm{emb}\;Q_{s}\right)}_{s\in S}$—this family then comprises all of the roots of $B:=A_{k}^{2}\equiv A_{s}^{2}\;\forall k,s$. Furthermore, by Lemma 23, there exists a cptp $A_{s}$ if and only if there exists a stochastic $Q_{s}$, and the reduction follows.

Finally, we can extend our reduction to the programs Doubly Stochastic Root and Doubly Stochastic Divisibility as well as Nonnegative Divisibility, defined analogously, see our comment in Corollary 27 and the complete reduction tree in Fig. 1. □

At this point, we observe the following fact.

Lemma 21 *All the above* Divisibility *and* Root *problems in* Definition 15, Definition 16, Definition 17, Definition 18, Definition 19 *are contained in* NP*.*

Proof It is straightforward to come up with a witness and a verifier circuit that satisfies the definition of the decision class NP. For example in the cptp case, a witness is a matrix root that can be checked to be a cptp map using Remark 13 and squared in polynomial time, which is the verifier circuit. Both circuit and witness are clearly poly-sized and hence the claim follows. □

By encoding an instance of 1-in-3sat into a family of nonnegative matrices ${\left(M_{s}\right)}_{s\in S}$, we show the implication 1-in-3sat⟶Nonnegative Root and 1-in-3sat⟶(Doubly) Stochastic/cptp Divisibility, accordingly, from which NP-hardness of (Doubly) Stochastic/cptp Divisibility follows. The entire chain of reduction can be seen in Fig. 1.

### 2.4 Reduction of Stochastic Root to CPTP Root

This reduction is based on the following embedding.

Definition 22 Let $\left\{e_{i}\right\}$ be an orthonormal basis of $K^{d}$. The embedding emb is defined as

$$\begin{array}{cc} \mathrm{emb}:K^{d\times d} & ↪K^{d^{2}\times d^{2}}\;\;,\\ A & ⟼B:=\sum_{i,j=1}^{d}A_{ij}(e_{i}⊗e_{i}){\left(e_{j}⊗e_{j}\right)}^{T}=\sum_{i,j=1}^{d}A_{ij}(e_{i}e_{j}^{T})⊗(e_{i}e_{j}^{T})\;\;. \end{array}$$

We observe the following.

Lemma 23 *We use the same notation as in* Remark 13*. Let* $A\in K^{d\times d}$ *and* B:=embA*. Then* **A** *is positive (nonnegative) if and only if the Choi matrix* $C_{B}$ *is positive (semi-)definite. Furthermore, the row sums of* **A** *are* 1*—i.e.* $\sum_{j=1}^{d}A_{ij}=1\;\forall j=1,\ldots ,d$*—if and only if* ${\mathrm{tr}}_{2}(C_{B})=1_{d}$*. In addition, the spectrum of* **B** *satisfies* σ(B)⊆σ(A)∪{0}*.*

Proof The first claim follows directly from the matrix representation of our operators. There, the *Choi* isomorphism is manifest as the reshuffling operation or partial transpose

$$\cdot^{\Gamma }:K^{d^{2}\times d^{2}}⟶K^{d^{2}\times d^{2}},{\left[(e_{i}e_{j}^{T})⊗(e_{i}e_{j}^{T})\right]}^{\Gamma }⟼(e_{i}e_{i}^{T})⊗(e_{j}e_{j}^{T})\;\;.$$

For more details, see e.g. [2].

The second statement follows from

$$\begin{array}{cc} {\mathrm{tr}}_{2}(C_{B}) & ={\mathrm{tr}}_{2}\left(\sum_{i,j=1}^{d}A_{ij}(e_{i}e_{j}^{T})⊗(e_{i}e_{j}^{T})\right)\\ & =\sum_{i,j=1}^{d}A_{ij}e_{i}e_{i}^{T}=\mathrm{diag}\left(\sum_{j=1}^{d}A_{1j},\ldots ,\sum_{j=1}^{d}A_{dj}\right)\;\;. \end{array}$$

The final claim is trivial. □

This remark immediately yields the following consequence.

Corollary 24 *For a family of stochastic matrices* ${\left(Q_{s}\right)}_{s\in S}$ *parametrized by the index set S, there exists a family of square matrices* ${\left(A_{s}\right)}_{s\in S}:={\left(\mathrm{emb}\;Q_{s}\right)}_{s\in S}$*, such that* ${\left(Q_{s}\right)}_{s\in S}$ *contains a stochastic matrix if and only if* ${\left(A_{s}\right)}_{s\in S}$ *contains a* cptp *matrix.*

### 2.5 Reduction of Nonnegative Root to Stochastic Root

The difference between Nonnegative Root and Stochastic Root is the extra normalization condition in the latter, see Definition 11. The following two lemmas show that this normalization does not pose an issue, so we can efficiently reduce the problem Nonnegative Root to Stochastic Root.

Lemma 25 *For a family of square matrices* ${\left(M_{s}\right)}_{s\in S}$ *parametrized by the index set* S*, all of which with at least one positive entry, there exists a family of square matrices* ${\left(Q_{s}\right)}_{s\in S}$ *such that* ${\left(M_{s}\right)}_{s\in S}$ *contains a nonnegative matrix if and only if* ${\left(Q_{s}\right)}_{s\in S}$ *contains a stochastic matrix and such that* $\mathrm{rank}\;Q_{s}=\mathrm{rank}\;M_{s}+2\;\forall s\in S$*. Furthermore,* ${\left(Q_{s}\right)}_{s\in S}$ *can be constructed efficiently from* ${\left(M_{s}\right)}_{s\in S}$*.*

Proof We explicitly construct our family ${\left(Q_{s}\right)}_{s\in S}$ as follows. Pick an s∈S and denote $M:=M_{s}$. Let *d* be the dimension of **M**. We first pick $a\in R^{+}$ such that $a\max_{ij}M_{ij}=1/2$^1^ and define

$$\begin{array}{cc} Q_{s}:= & \frac{1}{1764d}\left(\begin{array}{ccc} 1764aM+637 & 735-1260aM & 392-504aM\\ 735-1260aM & 900aM+1029 & 360aM\\ 392-504aM & 360aM & 144aM+1372 \end{array}\right)\\ \equiv & \frac{a}{d}AA^{T}⊗M+\frac{1}{d}\left(BB^{T}+CC^{T}\right)⊗1\;\;, \end{array}$$

where by sum of matrix **M** and scalar *x* we mean M+x1, $1:={\left(1\right)}_{1\leq i,j\leq d}\in R^{d\times d}$, and

$$A:={\left(1,-\frac{5}{7},-\frac{2}{7}\right)}^{T}\;\;,\;B:={\left(\frac{1}{6},\frac{1}{2},-\frac{2}{3}\right)}^{T}\;\;,\;C:=-\frac{1}{\sqrt{3}}{\left(1,1,1\right)}^{T}\;\;.$$

Observe that {A,B,C} form an orthogonal set—if one wishes, normalizing and pulling out the constant as eigenvalue to the corresponding eigenprojectors would work equally well.

By construction, $Q_{s}$ is nonnegative if and only if $M_{s}$ is. Since the row sums of $Q_{s}$ are always 1, $Q_{s}$ is stochastic if and only if $M_{s}$ is nonnegative, and the claim follows. □

Lemma 26 *Let the notation be as in* Lemma 25 *and write* $\sqrt{N}$ *for the set of roots of* **N***, see* Definition 8*. Assume* ${\left(M_{s}\right)}_{s\in S}=\sqrt{N}$ *for some* $N\in C^{d\times d}$*. Then there exists a* $P\in C^{d\times d}$*, such that* $Q_{s}^{2}=P\;\forall s\in S$ *and* ${\left(Q_{s}\right)}_{s\in S}\subset \sqrt{P}$*. Furthermore, the complement of* ${\left(Q_{s}\right)}_{s\in S}$ *in* $\sqrt{P}$ *does not contain any stochastic roots.*

Proof The first statement is obvious, since for all s∈S,

$$Q_{s}^{2}=\frac{a^{2}}{d^{2}}\frac{78}{49}AA^{T}⊗M_{s}^{2}+\frac{1}{d}\left(\frac{13}{18}BB^{T}+CC^{T}\right)⊗1=:P\;\;,$$

and hence clearly ${\left(Q_{s}\right)}_{s\in S}\subset \sqrt{P}$.

The last statement is not quite as straightforward—it is the main reason our carefully crafted matrix $Q_{s}$ has its slightly unusual shape. All possible roots of **P** are of the form

$$\sqrt{P}=\frac{a}{d}AA^{T}⊗\sqrt{N}\pm \frac{1}{d}\left(BB^{T}\pm CC^{T}\right)⊗1\;\;.$$

It is easy to check that none of the other sign choices yields any stochastic matrix, so the claim follows.^2^ □

Corollary 27 *The results of* Lemma 25, Lemma 26 *also hold for doubly stochastic matrices—observe that our construction of* $Q_{s}$ *is already doubly stochastic.*

### 2.6 Reduction of 1-in-3sat to Nonnegative Root

We now embed an instance of a boolean satisfiability problem, 1-in-3sat—see Definition 87 for details—into a family of matrices ${\left(M_{s}\right)}_{s\in S}$ in a way that there exists an *s* such that $M_{s}$ is nonnegative if and only if the instance of 1-in-3sat is satisfiable. The construction is inspired by [8].

We identify

(1) true⟷1,false⟷−1.

Denote with $(m_{i1},m_{i2},m_{i3})\in {\left\{\pm 1\right\}}^{3}$ the three boolean variables occurring in the *i*^th^ boolean clause, and let $m_{i}\in \{\pm 1\}$ stand for the single *i*^th^ boolean variable. Then 1-in-3sat translates to the inequalities

(2) $$-\frac{3}{2}\leq m_{i1}+m_{i2}+m_{i3}\leq -\frac{1}{2}\;\forall i=1,\ldots ,n_{c}\;\;.$$

Theorem 28 *Let* $\left(n_{v},n_{c},m_{i},m_{ij}\right)$ *be a 1-*in*-3*sat *instance. Then there exists a family of matrices* ${\left(M_{s}\right)}_{s\in S}$ *such that* $\exists s:M_{s}$ *nonnegative iff the instance is satisfiable.*

To prove this, we first need the following technical lemma.

Lemma 29 *Let* $\left(n_{v},n_{c},m_{i},m_{ij}\right)$ *be a 1-*in*-3*sat *instance. Then there exists a family of matrices* ${\left(C_{s}\right)}_{s\in S}$ *such that* ∃*s: the first* $n_{c}$ *on-diagonal* 4×4 *blocks of* $C_{s}$ *are nonnegative iff the instance is satisfiable. In addition, we have* $C_{s}^{2}=C_{t}^{2}\;\forall s,t$*. Furthermore,* ${\left(C_{s}\right)}_{s\in S}\subset \sqrt{C_{s}^{2}}$*, and the complement contains no nonnegative root.*

Proof For every boolean variable $m_{k}$, define a vector $v_{k}\in R^{d}$ such that their first $n_{c}$ elements are defined via

$${\left(v_{k}\right)}_{i}:=\left\{ \begin{array}{cc} 1 & m_{k}\text{ occurs in }i\text{th clause}\\ 0 & \text{otherwise}\;\;. \end{array}\right.$$

We will specify the dimension *d* later—obviously $d\geq n_{c}$, and the free entries are used to orthonormalize all vectors in the end. For now, we denote the orthonormalization region with $\overset{\to }{o}$. We further define the vectors $c_{1},c_{2}\in R^{d}$ to have all 1s in the first $n_{c}$ entries, i.e. $c_{1,2}=(1,\ldots ,1,{\overset{\to }{o}}_{1,2})$. Let then

(3) $$C_{s}^{\prime }:=c_{1}c_{1}^{T}⊗\left(\begin{array}{cc} 1 & 1\\ -1 & 1 \end{array}\right)+\frac{1}{2}c_{2}c_{2}^{T}⊗\left(\begin{array}{cc} 0 & 1\\ 1 & 0 \end{array}\right)+\sum_{k=1}^{n_{v}}p_{k}v_{k}v_{k}^{T}⊗\left(\begin{array}{cc} 0 & 1\\ -1 & 0 \end{array}\right)\;\;.$$

The variables $p_{k}$ denote a specific rescaled choice of the boolean variables $m_{i}$, which—in order to avoid degeneracy—have to be distinct, i.e. via

(4) $$p_{i}=\left(1-\frac{1}{N}-\frac{i}{Nn_{v}}\right)m_{i}\;\forall i=1,\ldots ,n_{v}\;\;.$$

The $p_{ij}$ are defined accordingly from the $m_{ij}$ and N∈N is large but fixed.

Let further

$$C_{s}:=\left(\begin{array}{cc} C_{s}^{\prime } & 0\\ 0 & 0 \end{array}\right)\in C^{d\times d}\;\;,$$

where we have used an obvious block notation to pad $C_{s}^{\prime }$ with zeroes, which will come into play later.

The on-diagonal 2×2 blocks of $C_{s}$ then encode the 1-in-3sat inequalities from equation (2)—demanding nonnegativity—as the set of equations

$$\frac{3}{2}+p_{i1}+p_{i2}+p_{i3}\geq 0\;\text{and}\;-\frac{1}{2}-p_{i1}-p_{i2}-p_{i3}\geq 0\;\;.$$

Note that we leave enough head space such that the rescaling in equation (4) does not affect any of the inequalities—see section 2.8 for details.

Observe further that the eigenvalues corresponding to each eigenprojector in the last term of equation (3) necessarily have opposite sign, otherwise we create complex entries. We will later rescale $C_{s}$ by a positive factor, under which the inequalities are invariant, so the first claim follows.

**Fig. 2:**
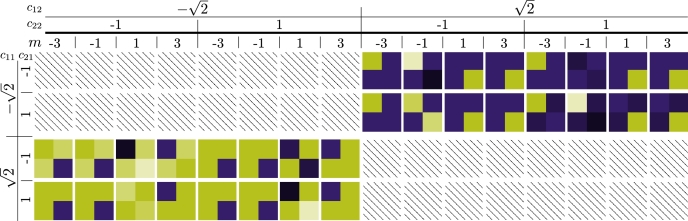
$C_{s}^{\prime }$ for various sign choices of the eigenvalues *c*_*ij*_, *i*,*j* = 1,2 corresponding to the eigenvectors *c*_1,2_. Only all positive signs and *m*:=∑_*j*_*m*_*ij*_ = −1 yields a nonnegative block (third from right in top row). Hatching signifies complex numbers, the colour scale is the same as in Fig. 3, i.e. light green denotes negative numbers, dark purple nonnegative entries.

We can always orthonormalize the vectors $c_{1,2}$ and $v_{k}$ using the freedom left in $\overset{\to }{o}$, hence we can achieve that $C_{s}^{2}=C_{t}^{2}\;\forall s,t$. It is straightforward to check that no other sign choice for the eigenvalues of the first two terms yields nonnegative blocks—see Fig. 2 for details. From this, the last two claims follow. □

### 2.7 Orthonormalization and handling the unwanted inequalities

As in [8], we have unwanted inequalities—the off-diagonal blocks in the first $4n_{c}$ entries and the blocks involving the orthonormalization region $\overset{\to }{o}$. We first deal with the off-diagonal blocks in favour of enlarging the orthonormalization region, creating more—potentially negative—entries in there, and then fix the latter.

*Off-diagonal blocks*. We begin with the following lemma.

Lemma 30 *Let the family* ${\left(C_{s}\right)}_{s\in S}$ *be defined as in the proof of* Lemma 29*, and* $\left(n_{v},n_{c},m_{i},m_{ij}\right)$ *the corresponding 1-*in*-3*sat *instance. Then there exists a matrix* $E\in C^{d\times d}$ *such that the top left* $4n_{c}\times 4n_{c}$ *block of* $C_{s}+E$ *has at least one negative entry* ∀*s iff the instance is not satisfiable. Furthermore,* $\mathrm{im}C_{s}⊥\mathrm{im}E\;\forall s$*, and* $C_{s}+E^{\prime }$ *has negative entries* ∀*s,* $\forall E^{\prime }\in \sqrt{E^{2}}\setminus \{E\}$*.*

Proof Define

$$E_{1}:=E_{1}E_{1}^{T}⊗\left(\begin{array}{cccc} 1 & 1 & 0 & 0\\ 1 & 1 & 0 & 0\\ 1 & 1 & 0 & 0\\ 1 & 1 & 0 & 0 \end{array}\right)\;\text{where}\;E_{1}:={\left(1,\ldots ,1,\overset{\to }{o}\right)}^{T}\;\;.$$

Then $E_{1}$ has rank 1.

From this mask, we now erase the first $n_{c}$ on-diagonal 4×4-blocks, while leaving all other entries in the upper left $4n_{c}\times 4n_{c}$ block positive. Define $b_{i}:=(e_{i},\overset{\to }{o})\in C^{d}$ for $i=1,\ldots ,n_{c}$ where $e_{i}$ denotes the *i*^th^ unit vector, and let

$$E:=\frac{7}{2}E_{1}-\frac{7}{2}\sum_{i=1}^{n_{c}}t_{i}b_{i}b_{i}^{T}⊗\left(\begin{array}{cccc} 1 & 1 & -1 & 0\\ 1 & 1 & -1 & 0\\ 0 & 0 & 0 & 0\\ 0 & 0 & 0 & 0 \end{array}\right)\;\;.$$

The variables $t_{i}$ are chosen close to 1 but distinct, e.g.

(5) $$t_{i}:=\left(1-\frac{1}{M}-\frac{i}{Mn_{c}}\right)\;\;,$$

where M∈N large but fixed. Then **E** has rank $n_{c}+1$, and adding **E** to $C_{s}$ trivializes all unwanted inequalities in the upper left $4n_{c}\times 4n_{c}$ block. By picking *M* large enough, the on-diagonal inequalities are left intact.

One can check that all other possible sign choices for the roots of **E** create negative entries in parts of the upper left block where $C_{s}$ is zero ∀*s*. Furthermore, $C_{s}$ and **E** have distinct nonzero eigenvalues by construction—the orthogonality condition is again straightforward, hence the last two claims follow. □

*Orthonormalization region*.

Lemma 31 *Let* 4n<d *and* δ≫1*. There exists a nonnegative rank* 2 *matrix* $D\in C^{d\times d}$ *such that the top left* 4n×4n *block of* **D** *has entries* $D_{ij}\in O(\delta^{-2})$ *if* j∤4 *and the rest of the matrix entries are* $\Omega (\delta^{-1})$*. If* $D^{\prime }\in \sqrt{D^{2}}$*, either the same holds true for* $D^{\prime }$*, or* $D_{ij}^{\prime }<0\;\forall j<4n+1,j|4$*.*

Proof Define

$$E_{2}:=(\underset{n\;\text{times}}{\underset{︸}{\frac{1}{\delta },\ldots ,\frac{1}{\delta }}},1,\ldots ,1)\in C^{d}$$

and let $E_{2}:=E_{2}E_{2}^{T}⊗1_{4}$, where $1_{4}:={\left(1\right)}_{1\leq i,j\leq 4}$. Let further

$$\Delta :=(\underset{n\;\text{times}}{\underset{︸}{\frac{1}{\delta },\ldots ,\frac{1}{\delta }}},-\frac{1}{\delta },\ldots ,-\frac{1}{\delta },a)\in C^{d}\;\;,$$

where 0<a<1 is used to orthonormalize Δ and $E_{2}$, which is the case if

$$a=-\frac{n}{\delta^{2}}+\frac{d-n-1}{\delta }\;\;.$$

**Fig. 3:**
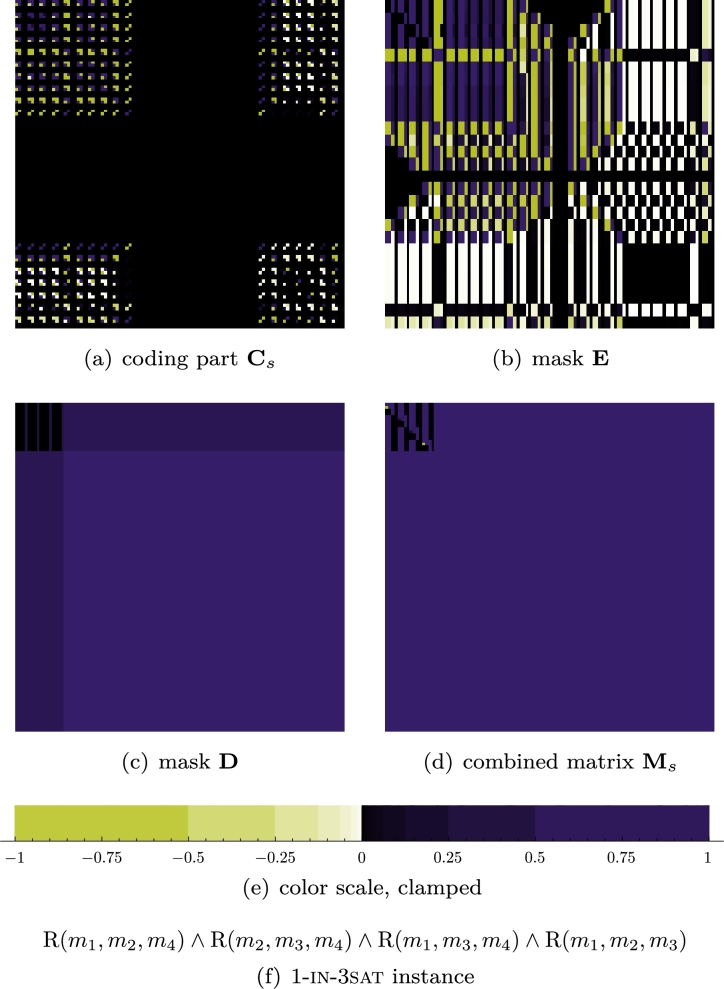
One branch of an unsatisfiable instance of 1-in-3sat encoded into a matrix of total rank 19. The negative entries—two bright dots—in the upper left block in the combined matrix (d) indicate that this branch does not satisfy the given instance. By looking at all other blocks, one sees that none translates to a nonnegative matrix. Observe that in this naïve implementation the orthonormalization region is suboptimally large.

By explicitly writing out the rank 2 matrix

$$D:=E_{2}\pm \Delta \Delta^{T}⊗\left(\begin{array}{cccc} 1 & 1 & 1 & 0\\ 1 & 1 & 1 & 0\\ 1 & 1 & 1 & 0\\ 1 & 1 & 1 & 0 \end{array}\right)\;\;,$$

it is straightforward to check that **D** fulfils all the claims of the lemma—see Fig. 3 for an example. □

### 2.8 Lifting singularities

The reader will have noted by now that even though we have orthonormalized all our eigenspaces, ensuring that the nonzero eigenvalues are all distinct, we have at the same time introduced a high-dimensional kernel in $C_{s}$, **E** and **D**. The following lemma shows that this does not pose an issue.

Lemma 32 *Let* ${\left(A_{s}\right)}_{s\in S}$ *be the family of primary rational roots of some degenerate* $B\in Q^{d\times d}$*. Then there exists a non-degenerate matrix* $B^{\prime }$*, such that for the family* ${\left(A_{s}^{\prime }\right)}_{s\in S}$ *of roots of* $B^{\prime }$*, we have* $A_{s}$ *positive iff* $A_{s}^{\prime }$ *positive. Furthermore, the entries of* $A_{s}^{\prime }$ *are rational with bit complexity* $r(A_{s}^{\prime })=O(\mathrm{poly}(r(A_{s})))$*.*

Proof Take a matrix $A\in {\left(A_{s}\right)}_{s\in S}$. We need to distort the zero eigenvalues $\left\{\lambda_{i}^{\left(0\right)}\right\}$ slightly away from 0. Using notation from Definition 36, a conservative estimate for the required smallness without affecting positivity would be

$$\lambda_{i}^{\left(0\right)}⟼\lambda_{i}^{\prime \;(0)}\;:\;0<\lambda_{i}^{\prime \;(0)}\leq \frac{1}{C\cdot d^{3}\cdot \max_{ij}\{|Z_{ij}|,|Z_{ij}^{-1}|\}}\;\;,$$

where we used the Jordan canonical form $A=Z\Lambda Z^{-1}$ for some invertible **Z** and $\Lambda =\mathrm{diag}(J_{0},J_{1})$, such that $J_{0}$ collects all Jordan blocks corresponding to the eigenvalue 0, and $J_{1}$ collects the remaining ones. □

This will lift all remaining degeneracies and singularities, without affecting our line of argument above. Observe that all inequalities in our construction were bounded away from 0 with enough head space independent of the problem size, so positivity in the lemma is sufficient.

We thus constructed an embedding of 1-in-3sat into non-derogatory and non-degenerate matrices, as desired. It is crucial to note that we do not lose anything by restricting the proof to the study of these matrices, as the following lemma shows.

Lemma 33 *There exists a Karp reduction of the* Divisibility *problems when defined for* all *matrices to the case of* non-degenerate and non-derogatory *matrices.*

Proof As shown in Lemma 21, containment in NP for this problem is easy to see, also in the degenerate or derogatory case. Since 1-in-3sat is NP-complete, there has to exist a poly-time reduction of the Divisibility problems—when defined for *all* matrices—to 1-in-3sat. Now embed this 1-in-3sat-instance with our construction. This yields a poly-time reduction to the non-degenerate non-derogatory case. □

### 2.9 Complete embedding

We now finally come to the proof of Theorem 28.

Proof of Theorem 28 Construct the family ${\left(C_{s}+E\right)}_{s\in S}$ using Lemma 29 and Lemma 30, ensuring that all orthonormalizing is done, which preliminarily fixes the dimension *d*. By Lemma 31, we now construct a mask D(δ) of dimension $d+d^{\prime }$, where $d^{\prime }>0$ is picked such that we can also orthonormalize all previous vectors with respect to $E_{2}$ and *δ*.

By Lemma 29, Lemma 30, Lemma 31, Lemma 32, the perturbed family ${\left(M_{s}^{\prime }\right)}_{s\in S}:={\left(C_{s}+E+ND(\delta )\right)}_{s}^{\prime }$—where *N* and δ∈Q are chosen big enough so that all unwanted inequalities are trivially satisfied—fulfils the claims of the theorem and the proof follows. □

We finalize the construction as follows. In Theorem 28, we have embedded a given 1-in-3sat instance into a family of matrices ${\left(M_{s}\right)}_{s\in S}$, such that the instance is satisfiable if and only if at least one of those matrices is nonnegative.

By rescaling the entire matrix such that $\max_{ij}{\left(M_{s}\right)}_{ij}=1/2$, we could show that this instance of 1-in-3sat is satisfiable if and only if the normalized matrix family ${\left(Q_{s}\right)}_{s\in S}$, which we construct explicitly, contains a stochastic matrix.

As shown in section 2.3, this can clearly be answered by Stochastic Divisibility, as the family ${\left(Q_{s}\right)}_{s\in S}$ comprises all the roots of a unique matrix **P**. If this matrix is *not* stochastic, our instance of 1-in-3sat is trivially not satisfiable. If the matrix *is* stochastic, we ask Stochastic Divisibility for an answer—a positive outcome signifies satisfiability, a negative one non-satisfiability.

### 2.10 Bit complexity of embedding

To show that our results holds for only polynomially growing bit complexity, observe the following proposition.

Proposition 34 *The bit complexity* $r(M_{s})$ *of the constructed embedding of a 1-*in*-3*sat *instance* $\left(n_{v},n_{c},m_{i},m_{ij}\right)$ *equals* $O(\mathrm{poly}(n_{v},n_{c}))$*.*

Proof We can ignore any construction that multiplies by a constant prefactor, for example Lemma 25 and Lemma 26. The renormalization for Lemma 25 to $\max_{ij}M_{s,ij}=1/2$ does not affect r either.

The rescaling in equation (4) and equation (5) yields a complexity of $O(\log n_{v})$, and the same thus holds true for Lemma 29 and Lemma 30.

The only other place of concern is the orthonormalization region. Let us write $a_{i}$ for all vectors that need orthonormalization. In the *n*^th^ step, we need to make up for O(n) entries with our orthonormalization, using the same amount of precision to solve the linear equations ${\left(a_{i}^{T}a_{n}=0\right)}_{1\leq i<n}$. This has to be done with a variant of the standard *Gauss* algorithm, e.g. the *Bareiss* algorithm—see for example [1]—which has nonexponential bit complexity.

Together with the lifting of our singularities, which has polynomial precision, we obtain $r(M_{s})=O(\mathrm{poly}(n_{v},n_{c}))$. Completing the embedding in section 2.9 changes the bit complexity by at most another polynomial factor, and hence the claim follows. □

## 3 Distribution divisibility

### 3.1 Introduction

Underlying stochastic and quantum channel divisibility, and—to some extent—a more fundamental topic, is the question of divisibility and decomposability of probability distributions and random variables. An illustrative example is the distribution of the sum of two rolls of a standard six-sided die, in contrast to the single roll of a twelve-sided die. Whereas in the first case the resulting random variable is obviously the sum of two uniformly distributed random variables on the numbers {1,…,6}, there is no way to achieve the outcome of the twelve-sided die as any sum of nontrivial “smaller” dice—in fact, there is no way of dividing *any* uniformly distributed discrete random variable into the sum of non-constant random variables. In contrast, a uniform continuous distribution can always be decomposed^3^ into two *different* distributions.

To be more precise, a random variable *X* is said to be *divisible* if it can be written as X=Y+Z, where *Y* and *Z* are non-constant independent random variables that are identically distributed (iid). Analogously, *infinite divisibility* refers to the case where *X* can be written as an infinite sum of such iid random variables.

If we relax the condition $Y\overset{d}{=}Z$—i.e. we allow *Y* and *Z* to have different distributions—we obtain the much weaker notion of *decomposability*. This includes using other sources of randomness, not necessarily uniformly distributed.

Both divisibility and decomposability have been studied extensively in various branches of probability theory and statistics. Early examples include *Cramer's theorem* [6], proven in 1936, a result stating that a Gaussian random variable can only be decomposed into random variables which are also normally distributed. A related result on $\chi^{2}$ distributions by *Cochran* [5], dating back to 1934, has important implications for the analysis of covariance.

An early overview over divisibility of distributions is given in [28]. Important applications of *n*-divisibility—the divisibility into *n* iid terms—is in modelling, for example of bug populations in entomology [18], or in financial aspects of various insurance models [30], [29]. Both examples study the overall distribution and ask if it is compatible with an underlying subdivision into smaller random events. The authors also give various conditions on distributions to be infinitely divisible, and list numerous infinitely divisible distributions.

Important examples for infinite divisibility include the *Gaussian*, *Laplace*, *Gamma* and *Cauchy* distributions, and in general all normal distributions. It is clear that those distributions are also finitely divisible, and decomposable. Examples of indecomposable distributions are *Bernoulli* and discrete uniform distributions.

However, there does not yet exist a straightforward way of checking whether a given discrete distribution is divisible or decomposable. We will show in this work that the question of decomposability is NP-hard, whereas divisibility is in P. In the latter case, we outline a computationally efficient algorithm for solving the divisibility question. We extend our results to weak-membership formulations (where the solution is only required to within an error *ϵ* in total variation distance), and argue that the continuous case is computationally trivial as the indecomposable distributions form a dense subset.

We start out in section 3.2 by introducing general notation and a rigorous formulation of divisibility and decomposability as computational problems. The foundation of all our distribution results is by showing equivalence to polynomial factorization, proven in section 3.3. This will allow us to prove our main divisibility and decomposability results in section 3.4 and 3.5, respectively.

### 3.2 Preliminaries

#### 3.2.1 Discrete distributions

In our discussion of distribution divisibility and decomposability, we will use the standard notation and language as described in the following definition.

Definition 35 Let (Ω,F,p) be a discrete *probability space*, i.e. Ω is at most countably infinite and the *probability mass function* p:Ω⟶[0,1]—or *pmf*, for short—fulfils $\sum_{x\in \Omega }p(x)=1$. We take the *σ-algebra* F to be maximal, i.e. $F=2^{\Omega }$, and without loss of generality assume that the *state space* Ω=N. Denote the *distribution* described by p with D. A *random variable* X:Ω⟶B is a measurable function from the sample space to some set B, where usually B=R.

For the sake of completeness, we repeat the following well-known definition of characteristic functions.

Definition 36 Let D be a discrete probability distribution with pmf p, and X∼D. Then

$$\phi_{X}(\omega ):=E(e^{i\omega X})=\int_{\Omega }e^{i\omega x}dF_{X}(x)=\sum_{x\in \Omega }p(x)e^{i\omega x}$$

defines the *characteristic function* of D.

It is well-known that two random variables with the same characteristic function have the same cumulative density function.

Definition 37 Let the notation be as in Definition 35. Then the distribution D is called *finite* if p(k)=0∀k≥N for some N∈N.

Remark 38 Let D be a discrete probability distribution with pmf p. We will—without loss of generality—assume that p(0)≠0 and p(k)=0∀k<0 for the pmf p of a finite distribution. It is a straightforward shift of the origin that achieves this.

#### 3.2.2 Continuous distributions

Definition 39 Let (X,A) be a *measurable space*, where A is the *σ*-algebra of X. The probability distribution of a *random variable X* on (X,A) is the *Radon–Nikodym derivative f*, which is a measurable function with $P(X\in A)=\int_{A}fd\mu$, where *μ* is a reference measure on (X,A).

Observe that this definition is more general than Definition 35, where the reference measure is simply the counting measure over the discrete sample space Ω. Since we are only interested in real-valued univariate continuous random variables, observe the following important remark.

Remark 40 We restrict ourselves to the case of X=R with A the *Borel* sets as measurable subsets and the *Lebesgue* measure *μ*. In particular, we only regard distributions with a *probability density function f*—or *pdf*, for short—i.e. we require the *cumulative distribution function* $P(x):=P(X\leq x)\equiv \int_{y\leq x}f(y)dy$ to be absolutely continuous.

Corollary 41 *The cumulative distribution function* P *of a continuous random variable X is almost everywhere differentiable, and any piecewise continuous function f with* $\int_{R}f(x)dx=1$ *defines a valid continuous distribution.*

#### 3.2.3 Divisibility and decomposability of distributions

To make the terms mentioned in the introduction rigorous, note the two following definitions.

Definition 42 Let *X* be a random variable. It is said to be *n*-*decomposable* if $X=Z_{1}+\ldots +Z_{n}$ for some n∈N, where $Z_{1},\ldots ,Z_{n}$ are independent non-constant random variables. *X* is said to be *indecomposable* if it is not decomposable.

Definition 43 Let *X* be a random variable. It is said to be *n*-*divisible* if it is *n*-decomposable as $X=\sum_{i=1}^{n}Z_{i}$ and $Z_{i}\overset{d}{=}Z_{j}\;\forall i,j$. *X* is said to be *infinitely* divisible if $X=\sum_{i=1}^{\infty }Z_{i}$, with $Z_{i}∼D$ for some nontrivial distribution D.

If we are not interested in the exact number of terms, we also simply speak of *decomposable* and *divisible*. We will show in section 3.5.7 that—in contrast to divisibility—the question of decomposability into more than two terms is not well-motivated.

Observe the following extension of Remark 38.

Lemma 44 *Let* D *be a discrete probability distribution with pmf* p*. If* p *obeys* Remark 38*, then we can assume that its factors do as well. In the continuous case, we can without loss of generality assume the same.*

Proof Obvious from positivity of convolutions in case of divisibility. For decomposability, we can achieve this by shifting the terms symmetrically. □

#### 3.2.4 Markov chains

To establish notation, we briefly state some well-known properties of *Markov* chains.

Remark 45 Take discrete iid random variables $Y_{1},\ldots ,Y_{n}∼D$ and write $P(Y_{i}=k)=p_{k}:=p(k)$ for all k∈N, independent of i=1,…,n. Define further

$$X_{i}:=\left\{ \begin{array}{cc} Y_{1}+\ldots +Y_{i} & i>0\\ 0 & \text{otherwise}\;\;. \end{array}\right.$$

Then $\left\{X_{n},n\geq 0\right\}$ defines a discrete-time *Markov chain*, since

$$\begin{array}{cc} P(X_{n+1}=k_{n+1}|X_{0}=k_{0}\wedge \ldots \wedge X_{n}=k_{n}) & =P(Y_{n+1}=k_{n+1}-k_{n})\\ & \equiv p_{k_{n+1}-k_{n}}\;\;. \end{array}$$

This last property is also called stationary independent increments, i.e. we add an iid random variable at each step.

Remark 46 Let the notation be as in Remark 45. The transition probabilities of the Markov chain are then given by

$$P_{ij}:=\left\{ \begin{array}{cc} p_{j-i} & j\geq i\\ 0 & \text{otherwise}\;\;. \end{array}\right.$$

In matrix form, we write the *transition matrix*

$$P:=\left(\begin{array}{cccc} p_{0} & p_{1} & p_{2} & \cdots \\ & p_{0} & p_{1} & \cdots \\ & & p_{0} & \cdots \\ & & & \ddots \end{array}\right)\;\;.$$

Working with transition matrices is straightforward—if the initial distribution is given by π:=(1,0,…), then obviously ${\left(\pi P\right)}_{i}=p_{i}$. Iterating **P** then yields the distributions of $X_{2},X_{3},\ldots$, respectively—e.g. ${\left(\pi P^{2}\right)}_{i}=P(X_{2}=i)\equiv P(Y_{1}+Y_{2}=i)$.

We know that $X_{2}$ is divisible—namely into $X_{2}=Y_{1}+Y_{2}$, by construction—but what if we ask this question the other way round? We will show in the next section that there exists a relatively straightforward way to calculate if an (infinite) matrix in the shape of **P** has a stochastic root—i.e. if D is divisible. Observe that this is not in contradiction with Theorem 1, as the theorem does not apply to infinite operators.

In contrast, the more general question of whether we can write a finite discrete random variable as a sum of nontrivial, potentially distinct random variables will be shown to be NP-hard.

### 3.3 Equivalence to polynomial factorization

Starting from our digression in section 3.2.4 and using the same notation, we begin with the following definition.

Definition 47 Denote with **S** the shift matrix $S_{ij}:=\delta_{i+1,j}$. Then we can write

$$P=p_{0}1+p_{1}S+p_{2}S^{2}+\ldots =\sum_{i=0}^{\infty }p_{i}S^{i}\in R_{\left[0,1\right]}[S]\;\;.$$

Since **S** just acts as a symbol, we write

$$f_{D}(x):=\sum_{i=0}^{N}p_{i}x^{i}\in R\;\text{where}\;R:=R_{\geq 0}[x]/∼\;\;,$$

and f∼g:⇔f=cg, c>0. We call $f_{D}$ the *characteristic polynomial* of D—not to be confused with the characteristic polynomial of a matrix. The equivalence space R defines the set of all characteristic polynomials, and can be written as

$$R=\overset{\infty }{\underset{i=n}{⋃}}R_{i}\;\text{where}\;R_{n}:=R/(x^{n})\;\;.$$

We mod out the overall scaling in order to keep the normalization condition $\sum_{k}p(k)=1$ implicit—if we write $f_{D}$, we will always assume $f_{D}(1)=1$. An alternative way to define these characteristic polynomials is via characteristic functions, as given in Definition 36.

Definition 48 $f_{D}(e^{i\omega })=\phi_{X}(\omega )$.

The reason for this definition is that it allows us to reduce operations on the transition matrix **P** or products of characteristic functions $\phi_{X}$ to algebraic operations on $f_{D}$. This enables us to translate the divisibility problem into a polynomial factorization problem and use algebraic methods to answer it. Observe that the $R_{N}$ are normed vector spaces, which we will make use of later.

Definition 49 We define norms on the space of characteristic polynomials of degree *N*—$R_{N}$—via ${\left\|f_{D}\right\|}_{N,p}:={\left\|{\left(p_{i}\right)}_{1\leq i\leq N}\right\|}_{\ell^{p}}$. If *N* is not explicitly specified, we usually assume $N=\deg f_{D}$.

First note the following proposition.

Proposition 50 *There is a* 1*-to-*1 *correspondence between finite distributions* D *and characteristic polynomials* $f_{D}$*, as defined in* Definition 47*.*

Proof Clear by Definition 48 and the uniqueness of characteristic functions. □

While this might seem obvious, it is worth clarifying, since this correspondence will allow us to directly translate results on polynomials to distributions.

The following lemma reduces the question of divisibility and decomposability—see Definition 42, Definition 43—to polynomial factorization.

Lemma 51 *A finite discrete distribution* D *is n-divisible iff there exists a polynomial* g∈R *such that* $g^{n}=f_{D}$*.* D *is n-decomposable iff there exist polynomials* $g_{1},\ldots ,g_{n}\in R$ *such that* $\prod_{i=1}^{n}g_{i}=f_{D}$*.*

Proof Assume that D is *n*-divisible, i.e. that there exists a distribution $D^{\prime }$ and random variables $Z_{1},\ldots ,Z_{n}∼D^{\prime }$ such that $X=\sum_{i=1}^{n}Z_{i}$. Denote with **Q** the transition matrix of $D^{\prime }$, as defined in Remark 46, and write q for its probability mass function. Then

$$P(X=j)=P\left(\sum_{i=1}^{n}Z_{i}=j\right)={\left(Q^{n}\pi \right)}_{j}\;\;,$$

as before. Write $g_{D^{\prime }}$ for the characteristic polynomial of $D^{\prime }$. By Definition 47, $g^{n}(S)\equiv f_{D}(S)$, and hence $g_{D^{\prime }}^{n}=f_{D}$. Observe that

$$1=\sum_{i}p(i)=f_{D}(1)\equiv g_{D^{\prime }}^{n}(1)={\left(\sum_{i}q(i)\right)}^{n}\;\;,$$

and hence $\sum_{i}q(i)=1$ is normalized automatically.

The other direction is similar, as well as the case of decomposability, and the claim follows. □

### 3.4 Divisibility

#### 3.4.1 Computational problems

We state an exact variant of the computational formulation of the question according to Definition 43—i.e. one with an allowed margin of error—as well as a weak membership formulation.

Definition 52 **${\text{Distribution Divisibility}}_{n}$**

**Instance.**: Finite discrete random variable X∼D.
**Question.**: Does there exist a finite discrete distribution $D^{\prime }:X=\sum_{i=1}^{n}Z_{i}$ for random variables $Z_{i}∼D^{\prime }$?

Observe that this includes the case n=2, which we defined in Definition 43.

Definition 53 **${\text{Weak Distribution Divisibility}}_{n,\epsilon }$**

**Instance.**: Finite discrete random variable X∼D with pmf $p_{X}(k)$.
**Question.**: If there exists a finite discrete random variable *Y* with pmf $p_{Y}(k)$, such that ${\left\|p_{X}-p_{Y}\right\|}_{\infty }<\epsilon$ and such that
  1. *Y* is *n*-divisible—return Yes
  2. *Y* is not *n*-divisible—return No.

#### 3.4.2 Exact divisibility

Theorem 54 ${\text{Distribution Divisibility}}_{n}\in P$*.*

Proof By Lemma 51 it is enough to show that for a characteristic polynomial $f\in R_{N}$, we can find a $g\in R:g^{n}=f$ in polynomial time. In order to achieve this, write ${\left(f\right)}^{1/n}$ as a Taylor expansion with rest, i.e.

$$\sqrt[{n}]{f(x)}=p(x)+R(x)\;\text{where}\;p\in R_{N/n},R\in R_{N}\;\;.$$

If R≡0, then g=p *n*-divides *f*, and then the distribution described by *f* is *n*-divisible. Since the series expansion is constructive and can be done efficiently—see [25]—the claim follows.

If the distribution coefficients are rational numbers, another method is to completely factorize the polynomial—e.g. using the *LLL* algorithm, which is known to be easy in this setting—sort and recombine the linear factors, which is also in O(poly(ordf)), see for example [13]. Then check if all the polynomial root coefficients are positive. □

We collect some further facts before we move on.

Remark 55 Let p be the probability mass function for a finite discrete distribution D, and write suppp={k:p(k)≠0}. If max⁡suppp−min⁡suppp=:w, then D is obviously not *n*-divisible for n>w/2, and furthermore not for any *n* that do not divide w,n<w/2. Indeed, D is not *n*-divisible if the latter condition holds for *either* max⁡suppp or min⁡suppp.

Remark 56 Let X∼D be an *n*-divisible random variable, i.e. $\exists Z_{1},\ldots ,Z_{n}∼D^{\prime }:\sum_{i=1}^{n}Z_{i}=X$. Then $D^{\prime }$ is unique.

Proof This is clear, because R[x] is a unique factorization domain. □

#### 3.4.3 Divisibility with variation

As an intermediate step, we need to extend Theorem 54 to allow for a margin of error *ϵ*, as captured by the following definition.

Definition 57 **${\text{Distribution Divisibility}}_{n,\epsilon }$**

**Instance.**: Finite discrete random variable X∼D with pmf $p_{X}(k)$.
**Question.**: Do there exist finite random variables $Z_{1},\ldots ,Z_{n}∼D^{\prime }$ with pmfs $p_{Z}(k)$, such that ${\left\|\underset{n\;\text{times}}{\underset{︸}{p_{Z}⁎\ldots ⁎p_{Z}}}-p_{X}\right\|}_{\infty }<\epsilon$?

Lemma 58 ${\text{Distribution Divisibility}}_{n,\epsilon }$ *is in* P*.*

Proof Let $f(x)=\sum_{i=0}^{N}p_{i}x^{i}$ be the characteristic polynomial of a finite discrete distribution, and ϵ>0. By padding the distribution with 0s, we can assume without loss of generality that N=deg⁡f is a multiple of *n*. A polynomial root—if it exists—has the form $g(x)=\sum_{i=0}^{N}a_{i}x^{i}$, where $a_{i}\geq 0\;\forall i$. Then

$$\begin{array}{cc} g{\left(x\right)}^{n} & ={\left(\ldots +a_{3}x^{3}+a_{2}x^{2}+a_{1}x+a_{0}\right)}^{n}\\ & =\ldots +((n-1)a_{1}^{2}+na_{0}^{n-2}a_{2})x^{2}+na_{0}^{n-1}a_{1}x+a_{0}^{n}\;\;. \end{array}$$

Comparing coefficients in the divisibility condition $f(x)=g{\left(x\right)}^{n}$, the latter translates to the set of inequalities

$$\begin{array}{cc} a_{0}^{n} & \in (p_{0}-\epsilon ,p_{0}+\epsilon )\\ na_{0}^{n-1}a_{1} & \in (p_{1}-\epsilon ,p_{1}+\epsilon )\\ (n-1)a_{1}^{2}+na_{0}^{n-2}a_{2} & \in (p_{2}-\epsilon ,p_{2}+\epsilon )\\ & \;\;\vdots \end{array}$$

Each term but the first one is of the form $h_{i}(a_{1},\ldots ,a_{i-1})+na_{0}^{n-i}a_{i}\in (p_{i}-\epsilon ,p_{i}+\epsilon )$, where $h_{i}\geq 0\;\forall i$ is monotonic. This can be rewritten as $a_{i}\in U_{\epsilon /na_{0}^{n-i}}((p-h_{i}(a_{1},\ldots ,a_{i-1}))/na_{0}^{n-i})$. It is now easy to solve the system iteratively, keeping track of the allowed intervals $I_{i}$ for the $a_{i}$.

If $I_{i}=\emptyset$ for some *i*, we return No, otherwise Yes. We have thus developed an efficient algorithm to answer ${\text{Distribution Weak Divisibility}}_{n,\epsilon }$, and the claim of Lemma 58 follows. □

Remark 59 Given a random variable *X*, the algorithm constructed in the proof of Lemma 58 allows us to calculate the closest *n*-divisible distribution to *X* in polynomial time.

Proof Straightforward, e.g. by using binary search over *ϵ*. □

#### 3.4.4 Weak divisibility

For the weak membership problem, we reduce ${\text{Weak Distribution Divisibility}}_{n,\epsilon }$ to ${\text{Distribution Divisibility}}_{n,\epsilon }$.

Theorem 60 ${\text{Weak Distribution Divisibility}}_{n,\epsilon }\in \text{P}$*.*

Proof Let D be a finite discrete distribution. If ${\text{Distribution Divisibility}}_{n,\epsilon }$ answers Yes, we know that there exists an *n*-divisible distribution *ϵ*-close to D. In case of No, D itself is not *n*-divisible, hence we know that there exists a non-*n*-divisible distribution close to D. □

#### 3.4.5 Continuous distributions

Let us briefly discuss the case of continuous distributions—continuous meaning a non-discrete state space X, as specified in section 3.2.2. Although divisibility of continuous distributions is well-defined and widely studied, formatting the continuous case as a computational problem is delicate, as the continuous distribution must be specified by a finite amount of data for the question to be computationally meaningful. The most natural formulation is the continuous analogue of Definition 43 as a weak-membership problem. However, we can show that this problem is computationally trivial.

First observe the following intermediate result.

Lemma 61 *Take* $f\in C_{c,b}^{+}$ *with* suppf⊂A∪B*, where* A:=[0,M],B:=[2M,3M]*,* $M\in R_{>0}$*. We claim that if f is divisible, then both* $f{|}_{A}$ *and* $f{|}_{B}$ *are divisible.*

Proof Due to symmetry, it is enough to show divisibility for $f{|}_{A}$. Assuming *f* is divisible, we can write f=r⁎r, i.e. $f(x)=\int_{R}r(x-y)r(y)dy$. It is straightforward to show that r(x)=0∀x<0. Define

(6) $$\overset{¯}{r}(x)=\left\{ \begin{array}{cc} r(x) & x\in A/2\\ 0 & \text{otherwise}\;\;, \end{array}\right.$$

where A/2:={a/2:a∈A}. Then

$$\begin{array}{cc} \left(\overset{¯}{r}⁎\overset{¯}{r})(x\right) & =\int_{R}\overset{¯}{r}(x-y)\overset{¯}{r}(y)dy\\ & =\int_{R}dy\left\{ \begin{array}{cc} r(x-y) & x-y\in A/2\\ 0 & \text{otherwise} \end{array}\right.\cdot \left\{ \begin{array}{cc} r(y) & y\in A/2\\ 0 & \text{otherwise}\;\;. \end{array}\right. \end{array}$$

**Fig. 4:**
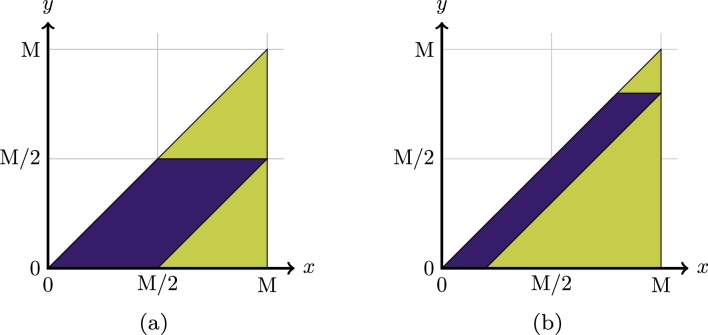
(a) Integration domains in Proposition 62 for $\overset{¯}{r}⁎\overset{¯}{r}$ (dark purple shading) and *f*|_A_ (light green shading), respectively. (b) Example for integration domains in Proposition 85 for $\overset{¯}{r}⁎\overset{¯}{s}$ (dark purple shading) and *f*|_A_ (light green shading), respectively.

We see that $(\overset{¯}{r}⁎\overset{¯}{r})(x)=0$ for x∉A. For x∈A, the support of the integrand is contained in $\{y:y\in x-A/2\wedge y\in A/2\}=x-A/2\cap A/2:=S_{x}$, and hence we can write $(\overset{¯}{r}⁎\overset{¯}{r})(x)=\int_{S_{x}}r(x-y)r(y)dy$. It hence remains to show that $f{|}_{A}(x)=\int_{S_{x}}r(x-y)r(y)dy\;\forall x\in A$. The integrand r(x−y)r(y)=0∀y<0∨y>x. The difference in the integration domains can be seen in Fig. 4. We get two cases.

Let x∈A. Assume $\exists y^{\prime }\in (M/2,M)$ such that $r(x-y^{\prime })r(y^{\prime })>0$. Let $x^{\prime }:=2y^{\prime }$. We then have $r{\left(y^{\prime }\right)}^{2}=r(x^{\prime }-y^{\prime })r(y^{\prime })>0$, and due to continuity $f(x^{\prime })>0$, contradiction, because $x^{\prime }\in (M,2M)$.

Analogously fix $x^{\prime }\in (M/2,M)$. Assume $\exists y^{\prime }\in (0,x^{\prime }-M/2)$ such that $r(x^{\prime }-y^{\prime })r(y^{\prime })>0$, and thus $r(x^{\prime }-y^{\prime })>0$, where $a:=x^{\prime }-y^{\prime }>M/2$, 2a∈(M,2M). Then $r{\left(a\right)}^{2}=r(2a-a)r(a)>0$, due to continuity f(2a)>0, again contradiction. □

Proposition 62 *Let* $C_{c,b}^{+}$ *denote the set of piecewise continuous nonnegative functions of bounded support. Then the set of* nondivisible *functions,* $I:=\{f:∄r\in C_{c,b}:f=r⁎r\}$ *is dense in* $C_{c,b}$*.*

Proof It is enough to show the claim for functions $f\in C_{c,b}^{+}$ with inf⁡suppf≥0. Let ϵ>0, and M:=sup⁡suppf. Take $j\in C_{c,b}$ to be nondivisible with suppj⊂(2M,3M), and define

$$g(x):=\left\{ \begin{array}{cc} f(x) & x<M\\ \epsilon j(x)/{\left\|j\right\|}_{\infty } & x\in (2M,3M)\\ 0 & \text{otherwise}\;\;. \end{array}\right.$$

By construction, ${\left\|f-g\right\|}_{\infty }<\epsilon$, but $g{|}_{\left(2M,3M\right)}\equiv j$ is not divisible, hence by Lemma 61 *g* is not divisible, and the claim follows. □

Corollary 63 *Let* ϵ>0*. Let X be a continuous random variable with pdf* $p_{X}(k)$*. Then there exists a nondivisible random variable Y with pdf* $p_{Y}(k)$*, such that* $\|p_{X}-p_{Y}\|<\epsilon$*.*

Proof Let ϵ>0 small. Since $C_{c,b}\subset \{f\;\text{integrable}\}=:L$, we can pick $f_{M}\in L:\mathrm{supp}\;f_{M}\in (-M,M),\|p_{X}-f_{M}\|<\epsilon /3$ and $\|f_{M}\|=1+\delta$ with |δ|≤ϵ/3. Then

$$\left\|p_{X}-\frac{f_{M}}{\left\|f_{M}\right\|}\right\|=\left\|p_{X}-\frac{f_{M}}{1+\delta }\right\|\leq \|p_{X}-f_{M}\|+\frac{\epsilon }{2}\|f_{M}\|\leq \epsilon \;\;,$$

and Proposition 62 finishes the claim. □

Corollary 64 *Any weak membership formulation of divisibility in the continuous setting is trivial to answer, as for all* ϵ>0*, there always exists a nondivisible distribution ϵ close to the one at hand. Similar considerations apply to other formulations of the continuous divisibility problem.*

#### 3.4.6 Infinite divisibility

Let us finally and briefly discuss the case of infinite divisibility. While interesting from a mathematical point of view, the question of infinite divisibility is ill-posed computationally. Trivially, discrete distributions cannot be infinitely divisible, as follows directly from Theorem 54. A similar argument shows that neither the *ϵ*, nor the weak variant of the discrete problem is a useful question to ask, as can be seen from Lemma 58, Theorem 60.

By the same arguments as in section 3.4.5, the weak membership version is thus easy to answer and therefore trivially in P.

### 3.5 Decomposability

#### 3.5.1 Computational problems

We define the decomposability analogue of Definition 52, Definition 53 as follows.

Definition 65 **Distribution Decomposability**

**Instance.**: Finite discrete random variable X∼D.
**Question.**: Do there exist finite discrete distributions $D^{\prime },D^{\prime \prime }:X=Z_{1}+Z_{2}$ for random variables $Z_{1}∼D^{\prime },Z_{2}∼D^{\prime \prime }$?

Definition 66 **${\text{Weak Distribution Decomposability}}_{\epsilon }$**

**Instance.**: Finite discrete random variable X∼D with pmf $p_{X}(k)$.
**Question.**: If there exists a finite discrete random variable *Y* with pmf $p_{Y}(k)$, such that ${\left\|p_{X}-p_{Y}\right\|}_{\infty }<\epsilon$ and such that
  1. *Y* is decomposable—return Yes
  2. *Y* is indecomposable—return No.

In this section, we will show that Distribution Decomposability is NP-hard, for which we will need a series of intermediate results. Requiring the support of the first random variable $Z_{1}$ to have a certain size, i.e. $|\mathrm{supp}(p_{D^{\prime }})|=m$, yields the following program.

Definition 67 **${\text{Distribution Decomposability}}_{m},\;m\geq 2$**

**Instance.**: Finite discrete random variable X∼D with $|\mathrm{supp}(p_{D})|>m$.
**Question.**: Do there exist finite discrete distributions $D^{\prime },D^{\prime \prime }:X=Z_{1}+Z_{2}$ for random variables $Z_{1}∼D^{\prime },Z_{2}∼D^{\prime \prime }$ and such that $|\mathrm{supp}(p_{D^{\prime }})|=m$?

We then define Distribution Even Decomposability to be the case where the two factors have equal support.

**Fig. 5:**
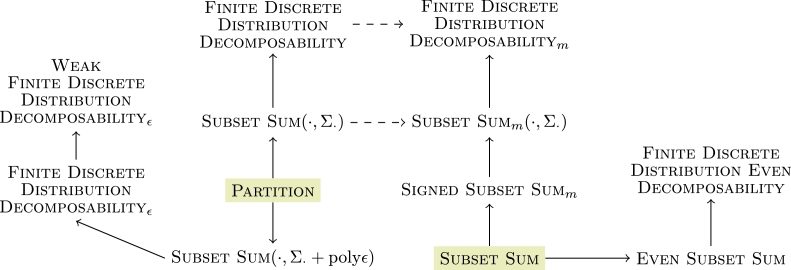
Complete chain of reduction for our discrete programs. The dashed lines are obvious and not mentioned explicitly.

The full reduction tree can be seen in Fig. 5.

Analogous to Lemma 21, we state the following observation.

Lemma 68 *All the above* Decomposability *problems in* Definition 65, Definition 66 *are contained in* NP*.*

Proof It is straightforward to construct a witness and a verifier that satisfies the definition of the decision class NP. For example in Definition 78, a witness is given by two tables of numbers which are easily checked to form finite discrete distributions. Convolving these lists and comparing the result to the given distribution can clearly be done in polynomial time. Both verification and witness are thus poly-sized, and the claim follows. □

#### 3.5.2 Even decomposability

We continue by proving that Distribution Even Decomposability is NP-hard. We will make use of the following variant of the well-known Subset Sum problem, which is NP-hard—see Lemma 92 for a proof. The interested reader will find a rigorous digression in section A.1.

Definition 69 **Even Subset Sum**

**Instance.**: Multiset S of reals with |S| even, l∈R.
**Question.**: Does there exist a multiset T⊊S with |T|=|S|/2 and such that $|\sum_{t\in T}t-\sum_{s\in S\setminus T}s|<l$?

This immediately leads us to the following intermediate result.

Lemma 70 Distribution Even Decomposability *is* NP*-hard.*

Proof Let (S,l) be an instance of Even Subset Sum. We will show that there exists a polynomial f∈R of degree 2|S| such that *f* is divisible into f=g⋅h with deg⁡g=deg⁡h iff (S,l) is a Yes instance. We will explicitly construct the polynomial f∈R. As a first step, we transform the Even Subset Sum instance (S,l), making it suited for embedding into *f*.

Let N:=|S| and denote the elements in S with $s_{1},\ldots ,s_{N}$. We perform a linear transformation on the elements $s_{i}$ via

(7) $$b_{i}:=a\left(s_{i}-\frac{1}{\left|S^{\prime }\right|}\sum_{s\in S^{\prime }}s\right)+\frac{al}{2|S^{\prime }|}\;\text{for}\;i=1,\ldots ,N\;\;,$$

where $a\in R_{>0}$ is a free scaling parameter chosen later such that $|b_{i}|<\delta \in R_{+}$ small. Let $B:=\{b_{1},\ldots ,b_{N}\}$. By Lemma 93, we see that Even Subset Sum(S,l)=Even Subset Sum(B,al). Since further $\sum_{i}b_{i}=al/2>0$, we know that (B,al) is a Yes instance if and only if there exist two non-empty disjoint subsets $B_{1}\cup B_{2}=B$ such that both

(8) $$\sum_{i\in B_{1}}b_{i}>0\;\text{and}\;\sum_{i\in B_{2}}b_{i}>0\;\;.$$

The next step is to construct the polynomial *f* and prove that it is divisible into two polynomial factors f=g⋅h if and only if (B,al) is a Yes instance. We first define quadratic polynomials $g(b_{i},x):=x^{2}+b_{i}x+1$ for i=1,…,N, and set $f_{T}(x):=\prod_{b\in T}g(b,x)$ for T⊂B. Observe that for suitably small *δ*, the $g(b_{i},x)$ are irreducible over R[x]. With this notation, we claim that $f_{B}(x)$ has the required properties.

In order to prove this claim, we first show that for sufficiently small scaling parameter *a*, a generic subset T⊂B with n:=|T| and $f_{T}(x)=:\sum_{i=1}^{2|T|}c_{i}x^{i}$, the coefficients $c_{i}$ satisfy

(9) $$c_{0}=1\;\;,$$

(10) $$\mathrm{sgn}(c_{1})=\mathrm{sgn}(\Sigma )\;\;,$$

(11) $$c_{2j}>0\;\text{for}\;j=1,\ldots ,|T|\;\;,$$

(12) $$\mathrm{sgn}(c_{2j+1})\geq \mathrm{sgn}(\Sigma )\;\text{for}\;j=1,\ldots ,|T|-1\;\;,$$

where $\Sigma :=\sum_{t\in T}t$. Indeed, if then $f_{B}=g\cdot h$, where g,h∈R, then $g=f_{B_{1}}$ and $h=f_{B_{2}}$ for aforementioned subsets $B_{1},B_{2}⊊B$, and conversely if (B,al) is a Yes instance, then $f_{B}=f_{B_{1}}\cdot f_{B_{2}}$—remember that R[x] is a unique factorization domain, so all polynomials of the shape $f_{T}$ necessarily decompose into quadratic factors.

By construction, $c_{0}=1$ and $c_{1}=n\Sigma$, so the first two assertions follow immediately. To address equation (11) and (12), we further split up the even and odd coefficients into

(13) $$c_{j}=:\left\{ \begin{array}{cc} c_{j,0}+c_{j,2}+\ldots +c_{j,j} & \text{if }j\text{ even}\\ c_{j,1}+c_{j,3}+\ldots +c_{j,j} & \text{if }j\text{ odd}\;\;, \end{array}\right.$$

where $c_{j,k}$ is the coefficient of $x^{j}b_{i_{1}}\cdots b_{i_{k}}$. We thus have $c_{j,k}=O(\delta^{k})$ in the limit δ→0—we will implicitly assume the limit in this proof and drop it for brevity. Our goal is to show that the scaling in *δ* suppresses the combinatorial factors, i.e. that $c_{j}$ is dominated by its first terms $c_{j,0}$ and $c_{j,1}$, respectively.

In order to achieve this, we need some more machinery. First regard $g(\delta ,x)=x^{2}+\delta x+1$. It is immediate that for an expansion

$$g{\left(\delta ,x\right)}^{n}=:\sum_{j=0}^{2n}x^{j}\sum_{k=0}^{n}d_{j,k}\delta^{k}\;\;,$$

we get coefficient-wise inequalities

(14) $$|c_{j,k}|\leq d_{j,k}\;\forall j=0,\ldots ,2n,k=0,\ldots ,n\;\;.$$

We will calculate the coefficients $d_{j,k}$ of $g{\left(\delta ,x\right)}^{n}$ explicitly and use them to bound the coefficients $c_{j,k}$ of $f_{T}(x)$.

Using a standard *Cauchy* summation and the uniqueness of polynomial functions, we obtain

$$\begin{array}{cc} g{\left(\delta ,x\right)}^{n} & =\sum_{j=0}^{n}\frac{1}{j!}{\left(1+x^{2}\right)}^{n-j}x^{j}{\left(n\right)}_{j}\delta^{j}\\ & =\sum_{j=0}^{n}\frac{\delta^{j}}{j!}{\left(n\right)}_{j}x^{j}\sum_{k=0}^{n-j}\left(\begin{array}{c} n-j\\ k \end{array}\right)x^{2k}\\ & \equiv \sum_{j=0}^{\infty }\sum_{k=0}^{\infty }\frac{\delta^{j}}{j!}{\left(n\right)}_{j}\left(\begin{array}{c} n-j\\ k \end{array}\right)x^{j+2k}\\ & =\sum_{j=0}^{\infty }\sum_{l=0}^{j}\frac{\delta^{l}}{l!}{\left(n\right)}_{l}\left(\begin{array}{c} n-l\\ j-l \end{array}\right)x^{2j-l}\\ & \equiv \sum_{j=0}^{n}\sum_{l=0}^{j}\frac{\delta^{l}}{l!}{\left(n\right)}_{l}\left(\begin{array}{c} n-l\\ j-l \end{array}\right)x^{2j-l}\\ & =\sum_{j=0}^{n}\sum_{l=j}^{2j}\frac{\delta^{2j-l}}{(2j-l)!}{\left(n\right)}_{2j-l}\left(\begin{array}{c} n-2j+l\\ l-j \end{array}\right)x^{l}\;\;. \end{array}$$

With ${\left(n\right)}_{l}$ we denote the falling factorial, i.e. ${\left(n\right)}_{l}=n(n-1)(n-2)\cdots (n-l+1)$. By convention, ${\left(n\right)}_{0}=1$.

Regarding even and odd powers of *x* separately, we can thus deduce that

$$\begin{array}{cc} g{\left(\delta ,x\right)}^{n} & =\sum_{j=0}^{2n}x^{j}\left\{ \begin{array}{cc} \sum_{k=0}^{\left\lfloor \frac{j}{2}\right\rfloor }\frac{\delta^{2k+1}}{(2k+1)!}\frac{{\left(n\right)}_{\lceil \frac{j}{2}\rceil +k}}{(\lfloor \frac{j}{2}\rfloor -k)!} & \text{if }j\text{ odd}\\ \sum_{k=0}^{\frac{j}{2}}\frac{\delta^{2k}}{(2k)!}\frac{{\left(n\right)}_{\frac{j}{2}+k}}{(\frac{j}{2}-k)!} & \text{if }j\text{ even} \end{array}\right.\\ & =\sum_{j=0}^{2n}x^{j}\left\{ \begin{array}{cc} {\left(n\right)}_{\left\lceil \frac{j}{2}\right\rceil }\sum_{k=0}^{\left\lfloor \frac{j}{2}\right\rfloor }\frac{\delta^{2k+1}}{(2k+1)!}\frac{{\left(n-\lceil \frac{j}{2}\rceil \right)}_{k}}{(\lfloor \frac{j}{2}\rfloor -k)!} & \text{if }j\text{ odd}\\ {\left(n\right)}_{\frac{j}{2}}\sum_{k=0}^{\frac{j}{2}}\frac{\delta^{2k}}{(2k)!}\frac{{\left(n-\frac{j}{2}\right)}_{k}}{(\frac{j}{2}-k)!} & \text{if }j\text{ even}\;\;. \end{array}\right. \end{array}$$

A straightforward estimate shows that for the even and odd case, we obtain the coefficient scaling

$$g{\left(\delta ,x\right)}^{n}=\sum_{j=0}^{2n}x^{j}\left\{ \begin{array}{cc} {\left(n\right)}_{\left\lceil \frac{j}{2}\right\rceil }\sum_{k=0}^{\left\lfloor \frac{j}{2}\right\rfloor }\delta^{2k+1}O(n^{k}) & \text{if }j\text{ odd}\\ {\left(n\right)}_{\frac{j}{2}}\sum_{k=0}^{\frac{j}{2}}\delta^{2k}O(n^{k}) & \text{if }j\text{ even}\;\;, \end{array}\right.$$

which means that e.g. picking $\delta =O(1/n^{2})$ is enough to exponentially suppress the higher order combinatorial factors.

We will now separately address the even and odd cases—equation (11) and (12).

*Even case*. As the constant coefficients $c_{j,0}=O(1)$ in *δ*, it is the same as for $g{\left(\delta ,x\right)}^{n}$ and by equation (14), we immediately get

$$\frac{\left|c_{j,2}+\ldots +c_{j,j}\right|}{c_{j,0}}=O(\delta )\;\;.$$

*Odd case*. Note that if Σ<0, we are done, so assume Σ>0 in the following. A simple combinatorial argument gives

$$c_{j,1}=\left(\begin{array}{c} n-1\\ (j-1)/2 \end{array}\right)\Sigma \;\;,$$

so it remains to show that $c_{j,1}>-c_{j,3}-\ldots -c_{j,j}$. Analogously to the even case, by equation (14), we conclude

$$\frac{\left|c_{j,3}+\ldots +c_{j,j}\right|}{c_{j,1}}=O(\delta )\;\;,$$

which finalizes our proof. □

#### 3.5.3 *m*-Support decomposability

In the next two sections we will generalize the last result to $\text{Distribution}{\text{Decomposability}}_{m}$. As a first observation, we note the following.

Lemma 71 *Let* f(n) *be such that* ${\left(f(n)\beta (f(n),n+1-f(n))\right)}^{-1}=O(\mathrm{poly}(n))$*. Then* ${\text{Distribution Decomposability}}_{f(|\cdot |)}\in \text{P}$*.*

Proof See proof of Theorem 54, and an easy scaling argument for $\left(\begin{array}{c} n\\ f(n) \end{array}\right)$ completes the proof. As in Remark 91, this symmetrically extends to $\text{Distribution}{\text{Decomposability}}_{\left|\cdot |-f(|\cdot |\right)}\in \text{P}$. □

Observe that f(n)=n/2 yields exponential growth, hence the remark is consistent with the findings in section 3.5.2.

We now regard the general case. As in the last section, we need variants of the Subset Sum problem, which are given in the following two definitions.

Definition 72 **${\text{Subset Sum}}_{m},\;m\in Z$**

**Instance.**: Multiset S of reals with |S| even, l∈R.
**Question.**: Does there exist a multiset T⊊S with |T|=m and such that $|\sum_{t\in T}t-\sum_{s\in S\setminus T}s|<l$?

Definition 73 **${\text{Signed Subset Sum}}_{m}$**

**Instance.**: Multiset S of positive integers or reals, x,y∈R:x≤y.
**Question.**: Does there exist a multiset T⊂S with |T|=m and such that $x<\sum_{t\in T}t-\sum_{s\in S\setminus T}s<y$?

Both are shown to be NP-hard in Lemma 90, Lemma 94, or by the following observation. In order to avoid having to take absolute values in the definition of ${\text{Subset Sum}}_{m}$, we reduce it to multiple instances of ${\text{Signed Subset Sum}}_{m}$, by using the following interval partition of the entire range (−l,l).

Remark 74 For every a>0,l>0, there exists a partition of the interval $\left(-l-2a,l+2a)={⋃}_{i=0}^{N-1}(x_{i},x_{i+1}\right)$ with suitable N∈N such that $x_{i+1}-x_{i}=2a$ and

$$(-l,l)=\left(\overset{N-2}{\underset{i=1}{⋃}}(x_{i},x_{i+1})\right)\setminus \left((x_{0},x_{1})\cup (x_{N-1},x_{N})\right)\;\;.$$

This finally leads us to the following result.

Lemma 75 ${\text{Distribution Decomposability}}_{m}$ *is* NP*-hard.*

Proof We will show the reduction ${\text{Distribution Decomposability}}_{m}⟵\text{Subset}{\text{Sum}}_{m}$. Let *m* be fixed. Let (S,l) be an Subset Sum instance. For brevity, we write $\Sigma_{S}:=\sum_{s\in S}s$. Without loss of generality, by Corollary 89, we again assume $\Sigma_{S}\geq 0$. Now define $a:=2(|S|l+2m\Sigma_{S}-|S|\Sigma_{S})/(2m-|S|)$. Using Remark 74, pick a suitable subdivision of the interval (−l−2a,l+2a), such that

$$\begin{array}{cc} {\text{Subset Sum}}_{m}(S,l)= & \left(\overset{N-2}{\underset{i=1}{⋁}}{\text{Signed Subset Sum}}_{m}(S,x_{i},x_{i+1})\right)\\ & \wedge \neg {\text{Signed Subset Sum}}_{m}(S,x_{0},x_{1})\\ & \wedge \neg {\text{Signed Subset Sum}}_{m}(S,x_{N-1},x_{N})\;\;. \end{array}$$

One can verify that

$$\begin{array}{cc} \text{Signed}\; & {\text{Subset Sum}}_{m}(S,x_{i}-a,x_{i}+a)\\ & ={\text{Signed Subset Sum}}_{m}(S+c(m,i),-\Sigma_{S+c(m,i)},\Sigma_{S+c(m,i)})\\ & ={\text{Subset Sum}}_{m}(S+c(m,i),\Sigma_{S+c(m,i)})\;\;, \end{array}$$

where we chose $c(m,i)=x_{i}/(2m-|S|)$. The latter program we can answer using the same argument as for the proof of Lemma 70, and the claim follows. □

As a side remark, this also confirms the following well-known fact.

Corollary 76 *Let* f(n) *be as in* Lemma 71*. Then* ${\text{Subset Sum}}_{f(|\cdot |)}\in \text{P}$*.*

#### 3.5.4 General decomposability

We have already invented all the necessary machinery to answer the general case.

Theorem 77 Distribution Decomposability *is* NP*-hard.*

Proof Follows immediately from Lemma 70, where we regard the special set of Subset Sum instances for which (S,l) is such that $l=\sum_{s\in S}s$. We show in Lemma 96 that $\text{Subset Sum}(\cdot ,\Sigma_{\cdot })$ is still NP-hard, thus the claim follows. □

#### 3.5.5 Decomposability with variation

As a further intermediate result—and analogously to Definition 57—we need to allow for a margin of error *ϵ*.

Definition 78 **${\text{Distribution Decomposability}}_{\epsilon }$**

**Instance.**: Finite discrete random variable X∼D with pmf $p_{X}(k)$.
**Question.**: Do there exist finite discrete random variables $Z_{1}∼D^{\prime },Z_{2}∼D^{\prime \prime }$ with pmfs $p_{Z_{1}}(k)$, $p_{Z_{2}}(k)$, such that ${\left\|p_{Z_{1}}⁎p_{Z_{2}}-p_{X}\right\|}_{\infty }<\epsilon$?

This definition leads us to the following result.

Lemma 79 ${\text{Distribution Decomposability}}_{\epsilon }$ *is* NP*-hard.*

Proof First observe that we can restate this problem in the following equivalent form. Given a finite discrete distribution D with characteristic polynomial $f_{D}$, do there exist two finite discrete distributions $D^{\prime },D^{\prime \prime }$ with characteristic polynomials $f_{D^{\prime }},f_{D^{\prime \prime }}$ such that ${\left\|f_{D}-f_{D^{\prime }}f_{D^{\prime \prime }}\right\|}_{d}<\epsilon$? Here, we are using the maximum norm from Definition 49, and assume without loss of generality that $\deg f_{D}=\deg f_{D^{\prime }}\deg f_{D^{\prime \prime }}$.

As $f_{D}$ is a polynomial, we can regard its *Viète* map $v:C^{n}⟶C^{n}$, where $n=\deg f_{D}$, which continuously maps the polynomial roots to its coefficients. It is a well-known fact—see [32] for a standard reference—that *v* induces an isomorphism of algebraic varieties $w:A_{k}^{n}/S_{n}\tilde{⟶}A_{k}^{n}$, where $S_{n}$ is the *n*^th^ symmetric group. This shows that $w^{-1}$ is polynomial, and hence the roots of $f_{D^{\prime }}f_{D^{\prime \prime }}$ lie in an O(ϵ)-ball around those of $f_{D}$. By a standard uniqueness argument we thus know that if $f_{D}=\prod_{i}f_{i}$ with $f_{i}=x^{2}+b_{i}x+1$ as in the proof of Lemma 70, then $f_{D^{\prime }}=\prod_{i}g_{i}$ with $g_{i}=a_{i}x^{2}+b_{i}^{\prime }x+c_{i}$, where $a_{i}=c_{i}=1+O(\epsilon )$, $b_{i}^{\prime }=b_{i}+O(\epsilon )$—we again implicitly assume the limit ϵ→0.

We continue by proving the reduction ${\text{Distribution Divisibility}}_{\epsilon }⟵\text{Subset}\text{Sum}(\cdot ,\Sigma_{\cdot }+\mathrm{poly}\;\epsilon )$, which is NP-hard as shown in Lemma 97. Let $S={\left\{s_{i}\right\}}_{i=1}^{N}$ be a Subset Sum multiset. We claim that it is satisfiable if and only if the generated characteristic function $f_{S}(x)$—where we used the notation of the proof of Lemma 70—defines a finite discrete probability distribution and the corresponding random variable *X* is a Yes instance for ${\text{Distribution Divisibility}}_{\epsilon }$.

First assume $f_{S}$ is such a Yes instance. Then $\sum_{s\in S}s\geq 0$, and there exist two characteristic polynomials $g=\prod_{i}g_{i}$ and $h=\prod_{i}h_{i}$ as above and such that ${\left\|f_{S}-gh\right\|}_{d}<\epsilon$. We also know that if $g_{i}=a_{i}x^{2}+b_{i}x+c_{i}$, then ∃T⊊S such that ${\left\{b_{i}\right\}}_{i}\in B_{\epsilon }(T)\subseteq R^{\left|T\right|}$, where T⊊S and $B_{\epsilon }(T)$ denotes an *ϵ* ball around the set T, and analogously for $h_{i}=a_{i}^{\prime }x^{2}+b_{i}^{\prime }x+c_{i}^{\prime }$, with ${\left\{b_{i}^{\prime }\right\}}_{i}\in B_{\epsilon }(S\setminus T)\subseteq R^{\left|S|-|T\right|}$. Regarding the linear coefficients, we thus have

(15) $$\begin{array}{cc} \left|\sum_{s\in S}s-\sum_{t\in T}t-\sum_{s\in S\setminus T}s\right| & =\left|\sum_{s\in S}s-\sum_{i=1}^{\left|T\right|}b_{i}-\sum_{i=1}^{\left|S\setminus T\right|}b_{i}^{\prime }+O(\epsilon )\right|\\ & \leq O(\epsilon )\leq \sum_{s\in S}s+O(\epsilon )\;\;. \end{array}$$

Now the case if $f_{S}$ is a No instance. Assume there exists a nontrivial multiset T⊊S satisfying

$$\left|\sum_{t\in T}t-\sum_{s\in S\setminus T}s\right|<\sum_{s\in S}s+O(\epsilon )\;\;.$$

Then by construction $\sum_{t\in T}t,\sum_{s\in S\setminus T}s\geq -O(\epsilon )$ and $f_{T}\cdot f_{S\setminus T}=f_{S}$, contradiction, and the claim follows. □

#### 3.5.6 Weak decomposability

Analogously to section 3.4.4, we now regard the weak membership problem of decomposability.

Theorem 80 ${\text{Weak Distribution Decomposability}}_{\epsilon }$ *is* NP*-hard.*

Proof In order to show the claim, we prove the reduction $\text{Weak Distribution}{\text{Decomposability}}_{\epsilon }⟵{\text{Distribution Decomposability}}_{g(\epsilon )}$, where the function g=O(ϵ). It is clear that the polynomial factor leaves the NP-hardness of the latter program intact.

We use the same notation as in the proof of Lemma 79. Let $f_{S}$ be a Yes instance of ${\text{Distribution Decomposability}}_{\epsilon }$, and define $S^{\prime }:=\{s+O(\epsilon ):s\in S\}$. From equation (15) it immediately follows that $f_{S^{\prime }}$ is a Yes instance of Distribution ${\text{Decomposability}}_{g(\epsilon )}$, where we allow g=O(ϵ). We have hence shown that there exists an O(ϵ) ball around each Yes instance that *solely* contains Yes instances.

A similar argument holds for the No instances. It is clear that these cases can be answered using ${\text{Weak Distribution Decomposability}}_{\epsilon }$, and the claim follows. □

#### 3.5.7 Complete decomposability

Another interesting question to ask is for the complete decomposition of a finite distribution D into a sum of indecomposable distributions. We argue that this decomposition is not unique.

Proposition 81 *There exists a family of finite distributions* ${\left(D_{n}\right)}_{n\in N}$ *with probability mass functions* $p_{n}(k):\max\mathrm{supp}\;p_{n}(k)=4n$ *and such that, for each* $D_{n}$*, there are at least n*! *distinct decompositions into indecomposable distributions.*

Proof We explicitly construct the family ${\left(D_{n}\right)}_{n\in N}$. Let n∈N. We will define a set of irreducible quadratic polynomials $\left\{p_{k},n_{k}\;\text{for}\;k=1,\ldots ,n\right\}$ such that $n_{k}$ are *not* positive, but $p_{k}n_{l}$ are positive quartics ∀k,l—and thus define valid probability distributions. Since R[x] is a unique factorization domain the claim then follows.

**Fig. 6:**
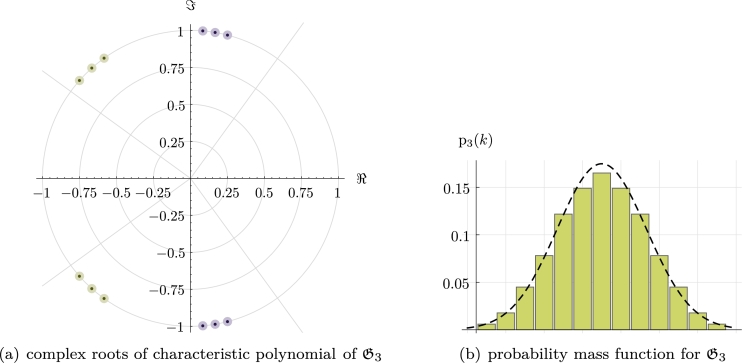
Construction of a family of distributions ${\left(D_{n}\right)}_{n\in N}$ with at least *n*! distinct decompositions, cf. Proposition 81. Shown is $D_{n}$ for *n* = 3. The dashed line shows a normal distribution for comparison.

Following the findings in the proof of Lemma 70, it is in fact enough to construct a set $\{a_{k},b_{k}:0<|a_{k}|<2,-2<b_{k}<0\;\text{for}\;k=1,\ldots ,n\}\subset R^{2n}$ and such that $a_{k}+b_{l}>0\;\forall k,l$—then let $p_{k}:=1+a_{k}x+x^{2}$, $n_{k}:=1+b_{k}x+x^{2}$. It is straightforward to verify that e.g.

$$a_{k}:=1+\frac{k}{2n}\;\text{and}\;b_{k}:=-\frac{k}{2n}$$

fulfil these properties. As an illustration, $D_{3}$ and the roots of its characteristic polynomial are shown in Fig. 6. □

Remark 82 Observe that for $b_{k}:=-k/2n^{2}$, the construction in Proposition 81 allows decompositions into *m* indecomposable terms, where m=n,…,2n.

Corollary 83 R *is not a unique factorization domain.*

Proposition 81 and Remark 82 show that an exponential number of complete decompositions—all of which have different distributions—do not give any further insight into the distribution of interest–indeed, as the number of positive indecomposable factors is not even unique. Asking for a non-maximal decomposition into indecomposable terms does therefore not answer more than whether the distribution is decomposable at all.

Indeed, the question whether one *can* decompose a distribution into indecomposable parts can be trivially answered with Yes, but if we include the condition that the factors have to be non-trivial, or for decomposability into a certain number of terms—say N≥2 or the maximum number of terms—the problem is also obviously NP-hard by the previous results.

In short, by Theorem 77, we immediately obtain the following result.

Corollary 84 *Let* D *be a finite discrete distribution. Deciding whether one can write* D *as any nontrivial sum of irreducible distributions is* NP*-hard.*

#### 3.5.8 Continuous distributions

Analogous to our discussion in section 3.4.5, the exact and *ϵ* variants of the decomposability question are computationally ill-posed. We again point out that answering the weak membership version is trivial, since the set of indecomposable distributions is dense, as the following proposition shows.

Proposition 85 *Let* $C_{c,b}^{+}$ *denote the piecewise linear nonnegative functions of bounded support. Then the set of* indecomposable *functions,* $J:=\{f:∄r,s\in C_{c,b}:f=r⁎s\}$ *is dense in* $C_{c,b}$*.*

Proof We first extend Lemma 61, and again take $f\in C_{c,b}^{+}:\mathrm{supp}\;f\subset A\cup B$. While not automatically true that r(x),s(x)=0∀x<0, we can assume this by shifting *r* and *s* symmetrically. We also assume inf⁡suppf=0, and hence inf⁡suppr=inf⁡supps=0—see Lemma 44 for details.

Since f(x)=0∀x∈(M,2M), we immediately get r(x)=s(x)=0∀x∈(M,2M). Furthermore, ∃m∈(0,M):r(x)=s(y)=0∀x∈(m,M],y∈(M−m,M]. Analogously to equation (6), we define

(16) $$\overset{¯}{r}(x)=\left\{ \begin{array}{cc} r(x) & x\in [0,m]\\ 0 & \text{otherwise} \end{array}\right.\;\text{and}\;\overset{¯}{s}(x)=\left\{ \begin{array}{cc} r(x) & x\in [0,M-m]\\ 0 & \text{otherwise}\;\;. \end{array}\right.$$

The integration domain difference is derived analogously, and can be seen in an example in Fig. 4. We again regard the two cases separately.

Let x∈A. Assume $\exists y^{\prime }\in (M-m,M)$ such that $r(x-y^{\prime })s(y^{\prime })>0$. Then $s(y^{\prime })>0$, contradiction. Now fix $x^{\prime }\in (m,M)$, and assume $\exists y^{\prime }\in (0,x^{\prime }-m):r(x^{\prime }-y^{\prime })s(y^{\prime })>0$. Since $x^{\prime }-y^{\prime }>x^{\prime }-x^{\prime }+m=m$, $r(x^{\prime }-y^{\prime })>0$ yields another contradiction.

The rest of the proof goes through analogously. □

Corollary 86 *Let* ϵ>0*. Let X be a continuous random variable with pmf* $p_{X}(k)$*. Then there exists an indecomposable random variable Y with pmf* $p_{Y}(k)$*, such that* $\|p_{X}-p_{Y}\|<\epsilon$*.*

Proof See Corollary 63. □

## 4 Conclusion

In section 2, we have shown that the question of existence of a stochastic root for a given stochastic matrix is in general at least as hard as answering 1-in-3sat, i.e. it is NP-hard. By Corollary 27, this NP-hardness result also extends to Nonnegative and Doubly Stochastic Divisibility, which proves Theorem 1. A similar reduction goes through for cptp Divisibility in Corollary 24, proving NP-hardness of the question of existence of a cptp root for a given cptp map.

In section 3, we have shown that—in contrast to cptp and stochastic matrix divisibility—distribution divisibility is in P, proving Theorem 4. On the other hand, if we relax divisibility to the more general decomposability problem, it becomes NP-hard as shown in Theorem 6. We have also extended these results to weak membership formulations in Theorem 5, Theorem 7—i.e. where we only require a solution to within *ϵ* in the appropriate metric—showing that all the complexity results are robust to perturbation.

Finally, in section 3.4.5 and 3.5.8, we point out that for continuous distributions—where the only computationally the only meaningful formulations are the weak membership problems or closely related variants—questions of divisibility and decomposability become computationally trivial, as the nondivisible and indecomposable distributions independently form dense sets.

As containment in NP for all of the NP-hard problems is easy to show (Lemma 21, Lemma 68), these problems are also NP-complete. Thus our results imply that, apart for the distribution divisibility problem which is efficiently solvable, all other divisibility problems for maps and distributions are equivalent to the famous P=NP conjecture, in the following precise sense: A polynomial-time algorithm for answering any one of these questions—(Doubly) Stochastic, Nonnegative or cptp Divisibility, or either of the Decomposability variants—would prove P=NP. Conversely, solving P=NP would imply that there exists a polynomial-time algorithm to solve all of these Divisibility problems.

## Appendix A

### A.1 NP-toolbox

*Boolean satisfiability problems*. Definition 87 1-in-3sat

**Instance:**: $n_{v}$ boolean variables $m_{1},\ldots ,m_{n_{v}}$ and $n_{c}$ clauses $R(m_{i1},m_{i2},m_{i3})$ where $i=1,\ldots ,n_{c}$, usually denoted as a 4-tuple $\left(n_{v},n_{c},m_{i},m_{ij}\right)$. The boolean operator R satisfies $$R(a,b,c)=\left\{ \begin{array}{cc} \text{True} & \text{if exactly one of }a,b\text{ or }c\text{ is True}\\ \text{False} & \text{otherwise}\;\;. \end{array}\right.$$
**Question:**: Does there exist a truth assignment to the boolean variables such that every clause contains exactly one true variable?

*Subset sum problems*. We start out with the following variant of a well-known NP-complete problem—see for example [11] for a reference.

Definition 88 Subset Sum, variant

**Instance.**: Multiset S of integer or rational numbers, l∈R.
**Question.**: Does there exist a multiset T⊊S such that $|\sum_{t\in T}t-\sum_{s\in S\setminus T}s|<l$?

From the definition, we immediately observe the following rescaling property. Corollary 89 *Let* a∈R∖{0} *and* (S,l) *a* Subset Sum *instance. Then* SubsetSum(S,l)=Subset Sum(aS,|a|l)*.*

For a special version of Subset Sum, ${\text{Subset Sum}}_{m}$—as defined in Definition 72—we observe the following. Lemma 90 ${\text{Subset Sum}}_{m}⟵\text{Subset Sum}$*.*
Proof If (S,l) is a Subset Sum instance, then

$$\text{Subset Sum}(S,l)=\overset{\left|S\right|}{\underset{m=1}{⋁}}{\text{Subset Sum}}_{m}(S,l)\;\;.$$

 □
Remark 91 It is clear that ${\text{Subset Sum}}_{m}(S,l)=\text{False}$ for |S|≤m∨0≥m. Furthermore, ${\text{Subset Sum}}_{m}(S,l)={\text{Subset Sum}}_{|S|-m}(S,l)$. Observe that this remark indeed makes sense, as ${\text{Subset Sum}}_{0}$ should give False, which is the desired outcome for m=|S|. We further reduce Subset Sum to Even Subset Sum, as defined in Definition 69. Lemma 92 Even Subset Sum⟵Subset Sum*.*
Proof Let (S,l) be an Subset Sum instance. Define $S^{\prime }:=S\cup \{0,\ldots ,0\}:|S^{\prime }|=2|S|$. Then if $\text{Even Subset Sum}(S^{\prime },l)=\text{True}$, we know that there exists $T^{\prime }\subset S^{\prime }:|\sum_{t\in T^{\prime }}t-\sum_{s\in S^{\prime }\setminus T^{\prime }}s|<l$. Let then $T:=T^{\prime }$ without the 0s. It is obvious that then $|\sum_{t\in T}t-\sum_{s\in S\setminus T}s|<l$. The False case reduces analogously, hence the claim follows. □

For Even Subset Sum, we generalize Corollary 89 to the following scaling property. Lemma 93 *Let* a∈R∖{0}*,* c∈R*, and* (S,l) *an* Even Subset Sum *instance. Then* Even Subset Sum(S,l)=Even Subset Sum(aS+c,|a|l)*, where addition and multiplication is defined element-wise.*
Proof Straightforward, since we require |S|=2|T|=2|S∖T|. □

For Definition 73, we finally show Lemma 94 ${\text{Signed Subset Sum}}_{m}⟵{\text{Subset Sum}}_{m}$*.*
Proof Immediate from ${\text{Signed Subset Sum}}_{m}(S,-l,l)={\text{Subset Sum}}_{m}(S,l)$. □

*Partition problems*. Another well-known NP-complete problem which will come into play in the proof of Theorem 77 is *set partitioning*. Definition 95 Partition

**Instance.**: Multiset A of positive integers or reals.
**Question.**: Does there exist a multiset T⊊A with $\sum_{t\in T}t=\sum_{s\in A\setminus T}s$?

Lemma 96 *For the special case of* Subset Sum *with instance* (S,l)*, where the bound* l∈R *equals the total sum of the instance numbers* $l=\sum_{s\in S}s$*, we obtain the equivalence* $\text{Subset Sum}(\cdot ,\Sigma_{\cdot })⟷\text{Partition}(\cdot )$*.*
Proof Let S be the multiset of a Subset Sum instance (S,l), where we assume without loss of generality that all S∋s≥0. Now first assume $\Sigma_{S}=0$. In that case the claim follows immediately, since the problems are identical. Without loss of generality, we can thus assume $\Sigma_{S}>0$ and regard the set $S^{\prime }:=S\cup \{-\Sigma_{S}/2,-\Sigma_{S}/2\}$, such that $\Sigma_{S^{\prime }}=0$. If now $\text{Subset Sum}(S^{\prime },0)=\text{True}$, we know that there exists $T⊊S^{\prime }:|\sum_{t\in T}t-\sum_{s\in S^{\prime }\setminus T}s|=0$. Now assume T contains both copies of $-\Sigma_{S}/2$. Then clearly

$$|\sum_{t\in T}t\;-\sum_{s\in S^{\prime }\setminus T}s|=|-\Sigma_{S}+\sum_{t\in T\setminus \{-\Sigma_{S}\}}t\;-\sum_{s\in S\setminus T}s|>0\;\;,$$

since |S∖T|>0. The same argument shows that exactly one $-\Sigma_{S}/2\in T,S\setminus T$, and hence Partition(S)=True. On the other hand, if Partition(S)=True, then it immediately follows that $\text{Subset Sum}(S,\Sigma_{S})=\text{True}$. □

Finally observe the following extension of Lemma 96. Lemma 97 *Let* ϵ>0*, f a polynomial. Then* $\text{Subset Sum}(\cdot ,\Sigma_{\cdot }+f(\epsilon ))⟷\text{Partition}(\cdot )$*.*
Proof The proof is the same as for Lemma 96, but we regard $S\cup \{-\Sigma_{S}/2-f(\epsilon )/2,-\Sigma_{S}/2-f(\epsilon )/2\}$ instead. □

## Footnotes

1 The exact bound is $a\max_{ij}M_{ij}\leq 43/81$.

2 The reader may try to find a simpler matrix that does the trick.

3 All continuous uniform distributions decompose into the sum of a discrete *Bernoulli* distribution and another continuous uniform distribution. This decomposition is never unique.

## References

[1] Bareiss E.H. (sep 1968). Sylvester's identity and multistep integer-preserving Gaussian elimination. Math. Comp..

[2] Bengtsson I., Życzkowski K. (2006). Geometry of Quantum States: An Introduction to Quantum Entanglement.

[3] Charitos T., de Waal P.R., van der Gaag L.C. (mar 2008). Computing short-interval transition matrices of a discrete-time Markov chain from partially observed data. Stat. Med..

[4] Choi M.-D. (1975). Completely positive linear maps on complex matrices. Linear Algebra Appl..

[5] Cochran W.G. (oct 1934). The distribution of quadratic forms in a normal system, with applications to the analysis of covariance. Math. Proc. Cambridge Philos. Soc..

[6] Cramér H. (dec 1936). Über eine Eigenschaft der normalen Verteilungsfunktion. Math. Z..

[7] Cubitt T.S., Eisert J., Wolf M.M. (mar 2012). Extracting dynamical equations from experimental data is NP hard. Phys. Rev. Lett..

[8] Cubitt T.S., Eisert J., Wolf M.M. (jan 2012). The complexity of relating quantum channels to master equations. Comm. Math. Phys..

[9] Egleston P.D., Lenker T.D., Narayan S.K. (mar 2004). The nonnegative inverse eigenvalue problem. Linear Algebra Appl..

[10] Elfving G. (1937). Zur Theorie der Markoffschen Ketten. Acta Soc. Sci. Fennicae N. Ser. A.

[11] Garey M.R., Johnson D.S. (jan 1979). Computers and Intractability: A Guide to the Theory of NP-Completeness.

[12] Gorini V. (aug 1976). Completely positive dynamical semigroups of N-level systems. J. Math. Phys..

[13] Hart W., van Hoeij M., Novocin A. (jun 2011). Practical polynomial factoring in polynomial time. Proceedings of the 36th International Symposium on Symbolic and Algebraic Computation – ISSAC '11.

[14] He Q.-M., Gunn E. (jun 2003). A note on the stochastic roots of stochastic matrices. J. Syst. Sci. Syst. Eng..

[15] Higham N.J. (apr 1987). Computing real square roots of a real matrix. Linear Algebra Appl..

[16] Higham N.J., Lin L. (aug 2011). On pth roots of stochastic matrices. Linear Algebra Appl..

[17] Jarrow R.A. (apr 1997). A Markov model for the term structure of credit risk spreads. Rev. Financ. Stud..

[18] S.K. Katti, Infinite divisibility of discrete distributions. III, 1977.

[19] Kingman J.F.C. (1962). The imbedding problem for finite Markov chains. Probab. Theory Related Fields.

[20] Lin L. (2011). Roots of stochastic matrices and fractional matrix powers.

[21] Lindblad G. (1976). On the generators of quantum dynamical semigroups. Comm. Math. Phys..

[22] Ljung L. (1987). System Identification: Theory for the User.

[23] Loring A.E. (1878). A Hand-Book of the Electromagnetic Telegraph.

[24] Minc H. (1988). Nonnegative Matrices.

[25] Müller N.T., Ottmann T. (jan 1987). Uniform computational complexity of Taylor series. Proceedings of the 13th International Colloquium on Automata, Languages and Programming.

[26] Müller-Hermes A., Reeb D., Wolf M.M. (2015). Quantum subdivision capacities and continuous-time quantum coding. IEEE Trans. Inform. Theory.

[27] Nielsen M.A., Knill E., Laflamme R. (nov 1998). Complete quantum teleportation using nuclear magnetic resonance. Nature.

[28] Steutel F., Kent J. (1979). Infinite divisibility in theory and practice. Scand. J. Stat..

[29] Thorin O. (jan 1977). On the infinite divisibility of the Pareto distribution. Scand. Actuar. J..

[30] Thorin O. (mar 1977). On the infinite divisibility of the lognormal distribution. Scand. Actuar. J..

[31] Waugh F.V., Abel M.E. (sep 1967). On fractional powers of a matrix. J. Amer. Statist. Assoc..

[32] Whitney H. (1972). Complex Analytic Varieties.

[33] Wolf M.M., Cirac J.I. (feb 2008). Dividing quantum channels. Comm. Math. Phys..

